# Orthogonal gap-enhanced Raman tags for interference-free and ultrastable surface-enhanced Raman scattering

**DOI:** 10.1515/nanoph-2021-0689

**Published:** 2022-03-28

**Authors:** Jin Li, Fugang Liu, Chang He, Feng Shen, Jian Ye

**Affiliations:** State Key Laboratory of Oncogenes and Related Genes, School of Biomedical Engineering, Shanghai Jiao Tong University, Shanghai 200030, P. R. China; Shanghai Key Laboratory of Gynecologic Oncology, Ren Ji Hospital, School of Medicine, Shanghai Jiao Tong University, Shanghai 200127, P. R. China; Institute of Medical Robotics, Shanghai Jiao Tong University, Shanghai 200240, P. R. China

**Keywords:** information security, nanogaps, orthogonal reporters, spectral interference, surface-enhanced Raman scattering

## Abstract

Spectral interference from backgrounds is not negligible for surface-enhanced Raman scattering (SERS) tags and often influences the accuracy and reliability of SERS applications. We report the design and synthesis of orthogonal gap-enhanced Raman tags (O-GERTs) by embedding alkyne and deuterium-based reporters in the interior metallic nanogaps of core–shell nanoparticles and explore their signal orthogonality as optical probes against different backgrounds from common substrates and media (e.g., glass and polymer) to related targets (e.g., bacteria, cancer cells, and tissues). Proof-of-concept experiments show that the O-GERT signals in the fingerprint region (200–1800 cm^−1^) are likely interfered by various backgrounds, leading to difficulty of accurate quantification, while the silent-region (1800–2800 cm^−1^) signals are completely interference-free. Moreover, O-GERTs show much higher photo and biological stability compared to conventional SERS tags. This work not only demonstrates O-GERTs as universal optical tags for accurate and reliable detection onto various substrates and in complex media, but also opens new opportunities in a variety of frontier applications, such as three-dimensional data storage and security labeling.

## 1 Introduction

Ultrahigh spectral sensitivity and specificity are the cornerstone of surface-enhanced Raman scattering (SERS) related applications [1], [2], [3], [4]. SERS tags based on their intrinsic fingerprint spectra have been widely employed as photostable probes for sensing, bioimaging, multiplex analysis, as well as security labeling [3, 5], [6], [7], [8]. In any scenario, background signals are not negligible and often overlap with signals of the tags in the fingerprint region (200–1800 cm^−1^). Background signals typically come from (1) the supporting substrates or media (e.g., glass, polymer, and biological buffer) in Raman measurements [9], [10], [11]; (2) the biological detection targets (e.g., bacteria, cells and tissues); (3) reporters’ spectral overlapping in multiplexing [12, 13]. Full avoidance of background overlap and interference in SERS is difficult, even using data analytics such as chemometrics [14], [15], [16]. This challenge highlights the need of designing SERS tags with the features of anti-interference and signal orthogonality against various backgrounds and the features of ultrahigh stability and sensitivity for prospective SERS applications, such as information security.

Recently, orthogonal SERS tags with unique Raman reporters (RRs) (such as alkyne and deuterium) have emerged to address above-mentioned issues in virtue to signal readouts in the Raman-silent region (1800–2800 cm^−1^) [6, 17, 18], where typical background species exhibit negligible vibrations. To date, a number of orthogonal SERS tags have been reported for sensing and bioimaging [12, 19], [20], [21], [22], [23]. However, orthogonal RRs are typically immobilized on the surface of plasmonic nanoparticles (NPs) or in the inter-nanogaps of NP dimers [18, 22, 24], [25], [26], which may suffer from poor stability or low sensitivity, thus limiting their widespread use in real applications. Herein, we report the design and synthesis of ultrastable core-gap-shell NPs with embedded orthogonal RRs, termed as orthogonal gap-enhanced Raman tags (O-GERTs), and explore their signal orthogonality as optical probes against different backgrounds including glass, polymers and biological samples. Through experiments in different application scenes, we demonstrate that these O-GERT signals in the fingerprint region are highly likely interfered by various backgrounds, while the silent region signals are completely separated from them. This proof-of-concept study opens the way towards the design of universal SERS nanotags for quantitative detection in complicated environments and demonstrates the potential for three-dimensional (3D) data storage and security labeling in resin protection layer.

## 2 Materials and methods

### 2.1 Materials and instruments

All chemicals were obtained commercially and used without further purification. Cetyltrimethylammonium chloride (CTAC, >98.0%), L-ascorbic acid (AA, >99.9%), 3-aminopropyltriethoxysilane (APTES), hydrochloric acid (HCl, 37 wt% in water), and sodium hydroxide were purchased from Sigma-Aldrich. Hydrogen tetrachloroaurate trihydrate (HAuCl_4_·3H_2_O) was purchased from Sinopharm Chemical Reagent Co. Ltd. (Shanghai, China). Glass (1 mm thick), and quartz wafer (0.5 mm thick) were purchased from Qiaoyi Biotechnology Co., Ltd. (Shanghai, China). 96-well plates were bought from Corning Incorporates (USA). PET (35 μm thick) was purchased from Deyi Adhesive Co. Ltd. (Shenzhen, China). Transparent photosensitive resin (product number 4200) was obtained from Nova Robotics, Inc. (Shenzhen, China). mPEG-SH (MW ∼5 kDa) was purchased from Xi’an Ruixi Biological Technology Co. Ltd. PBS, FBS, RPMI 1640 medium, and DMEM medium were bought from Gibco. Distilled water (18.0 MΩ cm) obtained from a Milli-Q Integral 5 system was used in all experiments. TEM images were recorded on the instruments of JEM-2100F (JEOL, Japan) with an accelerating voltage of 200 kV. The surface structure of O-GERT-cell mixtures coated on a silicon wafer was studied by a scanning electron microscope (SEM, FEI-QUANTA 200, Holland). UV–Vis spectra were measured with a UV1900 UV–Vis spectrophotometer (Aucybest, Shanghai, China).

### 2.2 Synthesis of O-GERTs

Gold (Au) nanoparticle (NP) cores with a diameter of 20 nm were first synthesized using a seed-mediated process described in our previous work [27]. The fabrication of O-GERTs involves two main steps as follows: (1) attaching to a monolayer of orthogonal reporters onto Au cores; (2) growth of Au shell with a desired thickness onto the core-reporter conjugates by adding Au salt (HAuCl_4_) and a mild reducing agent of AA [28, 29]. Brief, 100 μL of 4 mM *S*-(4-((4-(phenylethynyl)-2-(pyridin-4-yl)phenyl)ethynyl)phenyl)ethanethioate (SPPE) solution was added to Au cores (10 mL) under mild stirring at 25 °C for 3 h. These SPPE modified Au cores were then resuspended in 0.05 M CTAC after washing three times with 0.05 M CTAC solution. After that, 1 mL SPPE modified core solution was added into the mixed solution of 16 mL CTAC solution (0.05 M), 480 μL of AA (0.04 M), and 960 μL of HAuCl_4_ (4.86 mM) under vigorous sonication to afford O-GERTs in about 10 min. 2-Mercapto-4,5,6,7-*d*4-benzimidazole (MDBM) O-GERTs, *S*-(4-ethynylphenyl)ethanethioate (SEE) O-GERTs, *S*-(4-((trimethylsilyl)ethynyl)phenyl)ethanethioate (STE) O-GERTs and 4,4′-(ethyne-1,2-diyl)dibenzenethiol (EDBT) O-GERTs were prepared in a similar step.

### 2.3 FDTD calculations

The finite-difference time-domain (FDTD) simulations were carried out to analyze the electromagnetic field enhancement of the GERT and the Au NP. All the optical simulations were performed with the commercial software FDTD Solutions (Lumerical Inc., Canada) [28]. The empirical permittivity of Au was fitted with the multi-coefficient model (MCM). The surrounding medium was set as water that has a refractive index of 1.33 [30]. The refined mesh area with a smallest mesh size of 0.1 nm was utilized to cover the whole structure of O-GERT to guarantee the accuracy of simulations.

### 2.4 SERS measurements

Raman measurements were performed on a LabRAM Xplora INV system (Horiba, China). Normal Raman and SERS spectra were acquired with a confocal Raman microscope (785 nm, 10× objective, 29.8 mW, and 2 s of integration time). The SERS imaging for cancer tissue was carried out using 785 nm laser (29.8 mW) with an integration time of 100 ms per pixel. Unless otherwise specified, SERS spectra were performed on quartz slide substrates for spectral comparison. All the comparative experiments were done at a same particle concentration (0.2 nM).

### 2.5 Collection of polluted water samples

Three samples were collected from different sources. The sample 1 is composed of rhodamine 6G and methylene blue with 1:1 ratio as typical environmental pollutants in water [31, 32]. Sample 2 was obtained from laboratory wastewater in Room 301, School of Biomedical Engineering, Shanghai Jiao Tong University, Shanghai, China. Sample 3 was collected from sewage water in Room 302, No. 7, Lane 209, Guanshengyuan road, Xuhui district, Shanghai, China.

### 2.6 Bacteria/cells/tissues related experiments

*Clostridium bolteae* (CB) bacteria were obtained from Ruijin Hospital Affiliated to Shanghai Jiao Tong University School of Medicine (Shanghai, China). The O-GERT-bacterium sample was prepared by mixing 20 μL of SPPE O-GERTs (0.4 nM) with 100 μL of CB bacteria suspension with a total number of cells of 1.0 × 10^5^. HeLa cells were cultured at 37 °C in RPMI 1640 medium supplemented with 10% premium FBS and a 5% CO_2_ environment. The O-GERT-cell sample was prepared by mixing SPPE O-GERTs (0.4 nM, 20 μL) with 100 μL of HeLa cells suspension with total number of cells of 1.0 × 10^5^. Breast cancer tissues from a woman (age = 43) with breast lateral lump were provided by Ren Ji Hospital of Shanghai Jiao Tong University (Shanghai, China). All tissue experiments were approved by the Ethics Committee of Ren ji Hospital, School of Medicine, Shanghai Jiao Tong University.

### 2.7 Preparation of AuNP dimers linked by EDBT (EDBT AuNP dimers)

Uniform 30 nm AuNPs were prepared according to our previous work [26, 33]. AuNP dimers were prepared by the following steps. Glass slides (22 × 8 mm) were first treated with piranha solution containing 98% H_2_SO_4_ and 30% H_2_O_2_ (3:1 v/v) for 30 min. After thoroughly washing with ultrapure water and ethanol by sonication, the glass slides were placed in an APTES (10:1 v/v) ethanol solution for 30 min to produce amine-coated surfaces. These slides were cleaned with ethanol and ultrapure water by sonication, and then immersed in a solution of 30 nm AuNPs (30 nm, 0.1 nM) for 4 h. Once finished, the excess amine groups were etched from the glass surfaces using a solution of NaOH (2 mM) for 2 h. Then, the immobilized AuNPs were coated with EDBT reporter (200 μM) dissolved in a mixture solution of water and ethyl alcohol (1:1 v/v). The excess reporter EDBT was then removed, and another round of 30 nm AuNPs (1.2 nM) were added and pulled down onto the substrate *via* Au–S bonds, producing AuNP dimers on the substrate. Afterward, the assembled dimers were desorbed from the substrate by ultrasonication. Before use, the resultant solution underwent centrifugation at 8000 rpm for 4 min to purify the dimers, and then the precipitates were re-dispersed in aqueous solution containing thiol-PEG of 100 μM for stabilization of AuNP dimers.

### 2.8 Photostability experiments

SERS photostability measurements of both types of tags (AuNP tags and O-GERTs) were performed on solid GERTs or AuNPs dropped on a silicon substrate after drying under continuous laser (785 nm) irradiation with two different laser power densities: 2.95 × 10^5^ and 1.17 × 10^6^ W/cm^2^ for 30 min with an integration time of 100 ms per spectrum. Under the condition of such high laser power density, these tests are more challenging than most in biological environment SERS tags applied in, such as buffer media and biological tissue, where the environmental media absorbs a part of light and tags can move freely at certain condition. Brief, 60 μL samples (0.2 nM) were dripped on a silicon substrate and dried before measurement. Continuous irradiation with a 785 nm laser was applied to both types of tags. Quantitative analysis of photostability behaviors of both tags was performed with the integrated peak areas of Raman bands at 2196 cm^−1^ for SPPE O-GERTs, 2201 cm^−1^ for AuNP-SPPE tags, 2294 cm^−1^ for MDBM O-GERTs and 2297 cm^−1^ for AuNP-MDBM tags. Photo-bleaching time constant was obtained by fitting the SERS decay curves to equation *I* = *Ae*^(−*t*/*τ*)^ after evaluation of three different locations on the substrate [34, 35].

### 2.9 Biological stability experiments

For biological stability, both types of tags were conjugated with a protective layer of thiol-PEG for stabilization of plasmonic NPs in biofluids, and the PEGylation process was as follows according to the literature [5]. In brief, the reporter solutions (SPPE or MDBM, 4 mM) were first mixed with the as-prepared AuNPs for 1 h to form AuNP-reporter conjugates *via* Au–S bonds. Then a mPEG-SH solution (1 mM) was added dropwise to the above solutions and incubated for 3 h to afford the pegylated AuNP tags. The molar ratio of reporter and mPEG-SH to AuNPs was adjusted for maximal SERS intensities and minimal colloid aggregation. PEGylation of O-GERTs was prepared through adding mPEG-SH solution (1 mM) to the as-prepared O-GERT solution, and incubated for 3 h to afford the pegylated SPPE O-GERTs and MDBM O-GERTs. The molar quantities of mPEG-SH in pegylated O-GERTs and AuNP tags remain same. The obtained pegylated tags were diluted and stored at 4 °C for further use. Biological stability was assessed by incubating both types of tags in PBS, FBS, DMEM fluids for 100 h at 37 °C.

### 2.10 Manufacture of security labels by SlipChip microfluidic technique

SlipChip microfluidic molding method is a well-established one to produce micropatterns with fast and easy loading-slipping-solidification operations. In general, a SlipChip-based microfluidic device consisting of two plates with fluidic ducts and microcavities imprinted on the contacting surfaces. The top and bottom plates are assembled so that microcavities and fluidic ducts on the different plates partially overlap, thus establishing a continuous fluidic path. The more details about the SlipChip-based microfluidic can be found elsewhere [36]. The fabrication procedure of the resin embedded with O-GERTs includes four steps as follows. Brief, the SPPE O-GERTs were first well mixed with liquid resin (step 1). Following the mixture process, the mixed suspension was introduced into the slip molding device through a fluidic inlet by using positive pressure from pipetting. After the fluidic channel was filled completely, the top plate was manually moved up relative to the bottom plate, the fluidic path was broken apart, and the microcavities were isolated to form the compartments (step 2). Next, the raw material in the microcavities can be solidified after photo-crosslinking *via* photochemical reactions (step 3). Finally, the clamping fixture was removed, and the top plate and the bottom plate were separated to obtain the solidified labels with “SJTU” micropatterns from the micro-cavities using tweezers (step 4). The resin embedded with O-GERTs show great potential as a SERS-based security label with interference-free readouts.

### 2.11 Readout of security labels

The security label with “SJTU” micropattern was read *via* a confocal Raman system with a pinhole value of 300 μm and a resolution of 52 × 19 pixels under DuoScan mode. A 785 nm laser (3 × 10^5^ W/cm^2^, ×|10|× objective lens) was used with a 100 ms exposure time per pixel. The *x*–*y*–*z* SERS mapping image was obtained by combining multiple *x*–*y* SERS images taken at various *z* values with an interval of 5 μm. To obtain *x*–*y* SERS maps at targeted *z* values, we first focused the laser onto the substrate surface (bottom of the label), defined as “0” in *z* direction, and the certain distance was tuned.

### 2.12 Digitization of security labels

The Raman spectrum process flow, which is composed of preprocessing, Raman intensity extraction and digitization, was realized by MATLAB R2019a. (i) Preprocessing. A moving average method with a window size of 5 and an order of 3 was employed to smooth Raman spectra for restraining noises. Then the baseline correction was accomplished by the algorithm of adaptive iteratively reweighted penalized least squares (airPLS) (order = 3, lambda = 100). (ii) Raman intensity extraction. The Raman intensity of 2196 cm^−1^ of SPPE O-GERTs were then obtained and each raw represents the intensities of SPPE O-GERTs at the pixel. (iii) Digitization. Since the Raman intensity will be influenced by instruments and environmental conditions, all intensities will be normalized with *Z*-score method by columns. Then the multi-start optimization algorithm was performed to search a common globally optimal threshold for mapping the intensity of each pixel to an element of the set {0, 1, 2, 3}. The aim of multi-start optimization algorithm is to find the best threshold to obtain highest reproducibility for the same label. After finding the threshold, we calculated the similarity index (*I*) of the label. The similarity index (*I*) is defined as:

$$I=\frac{\text{The}\;\text{number}\;\text{of}\;\text{same}\;\text{pixels}\;\text{between}\;\text{two}\;\text{measurements}}{\text{The}\;\text{number}\;\text{of}\;\text{pixels}\;\text{in}\;\text{single}\;\text{measurement}}$$

### 2.13 TEM observation of NPs distribution inside resins

The label based on resin embedded SPPE O-GERTs was embedded in EPOM812 and polymerized in the oven at 60 °C for 48 h. Ultrathin sections of approximately 150 nm thick were cut with a diamond knife on a Leica UC6 ultra-microtome and transferred to the copper grid for TEM observations.

## 3 Results and discussion

### 3.1 Molecule selections for O-GERTs

**Figure 1:**
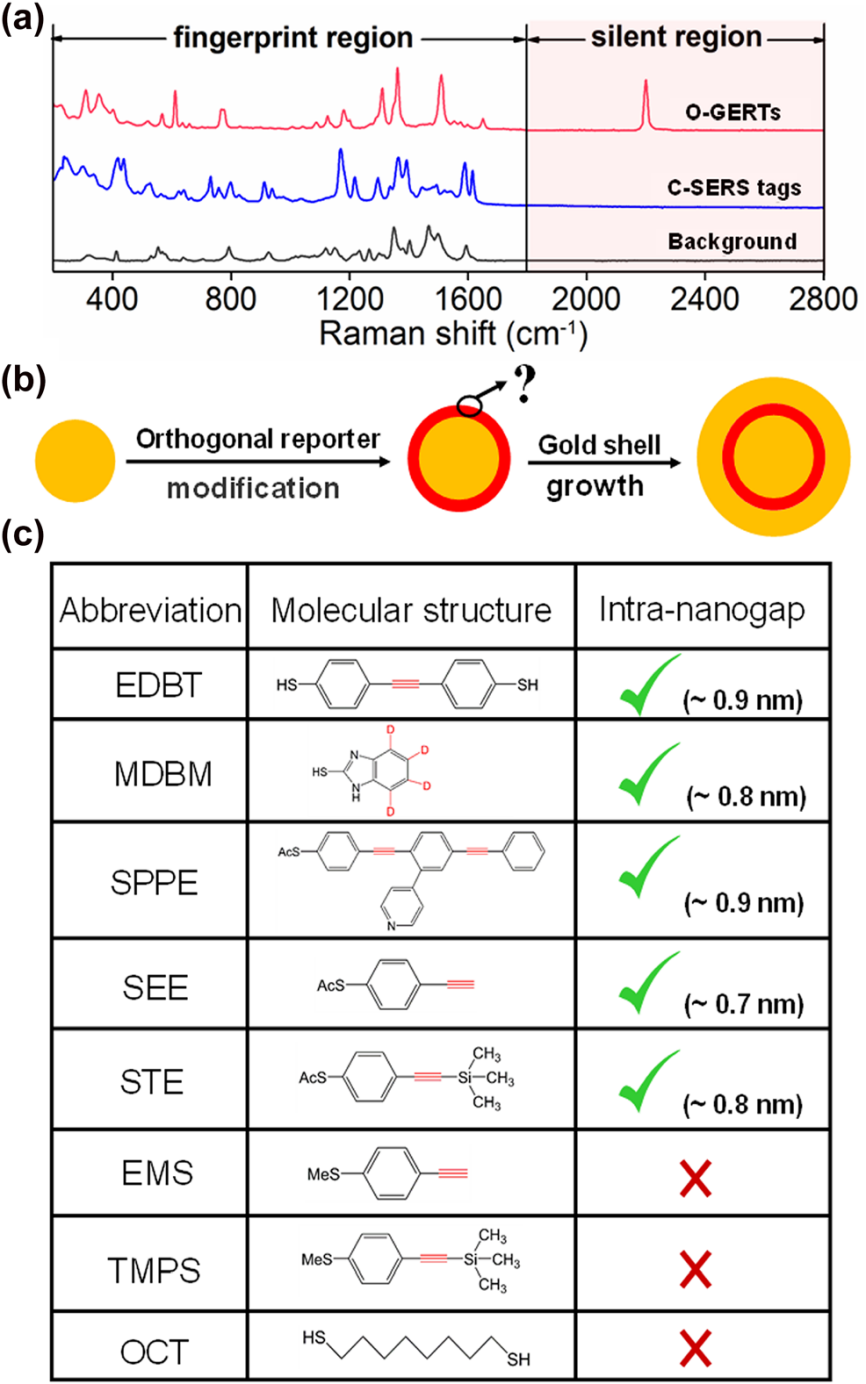
Design and synthesis of O-GERTs. (a) Schematic diagram of Raman spectra of O-GERTs, conventional SERS (C-SERS) tags and background. (b) Schematic diagram of synthesis of O-GERTs. (c) Selectivity of RRs for O-GERTs. Orthogonal groups are marked in red.

Orthogonal RRs exhibit signal readouts in the Raman-silent region (1800–2800 cm^−1^), where conventional SERS (C-SERS) tags and background species exhibit negligible vibrations (Figure 1a). We started this study by selecting orthogonal RRs for fabricating O-GERTs with intra-nanogaps given that the RR is a key component to determine the morphology of the nanogap and the Raman enhancement of the tags. A typical wet-chemistry approach consisting of two stages was employed to embed orthogonal RRs into metallic intra-nanogaps (Figure 1b): (1) attachment of a monolayer of orthogonal RRs onto gold (Au) cores, and (2) growth of Au shell with a desired thickness onto the core-RR conjugates by adding Au salt and a mild reduction agent of L-ascorbic acid. Seven kinds of orthogonal molecules (Scheme S1, Figures S1–S6) were evaluated for offering a rule on molecular selectivity of orthogonal RRs in O-GERTs (Figure 1c). TEM results indicate that the RRs with sulfhydryl or acetyl sulfide can be embedded in metallic intra-nanogaps due to high affinity towards Au, while the RRs with methylthio group are not able to form uniform intra-nanogaps (Figures S7a–c and S8). As a control molecule, 1,8-octanedithiol, with sulfhydryl groups and a flexible backbone chain, does not form intra-nanogaps either (Figure S7d). Therefore, a preliminary selection rule of orthogonal RRs to form O-GERTs with uniform nanogaps can be summarized that RRs prefer to have: (1) strong affinity between molecules and Au cores is necessary, e.g., molecules bearing sulfhydryl or acetyl sulfide; (2) a rigid molecular skeleton (e.g., aromatic ring) that may produce effective occupied area on the surface of Au cores (Figure 1c).

### 3.2 Characterization of O-GERTs

**Figure 2:**
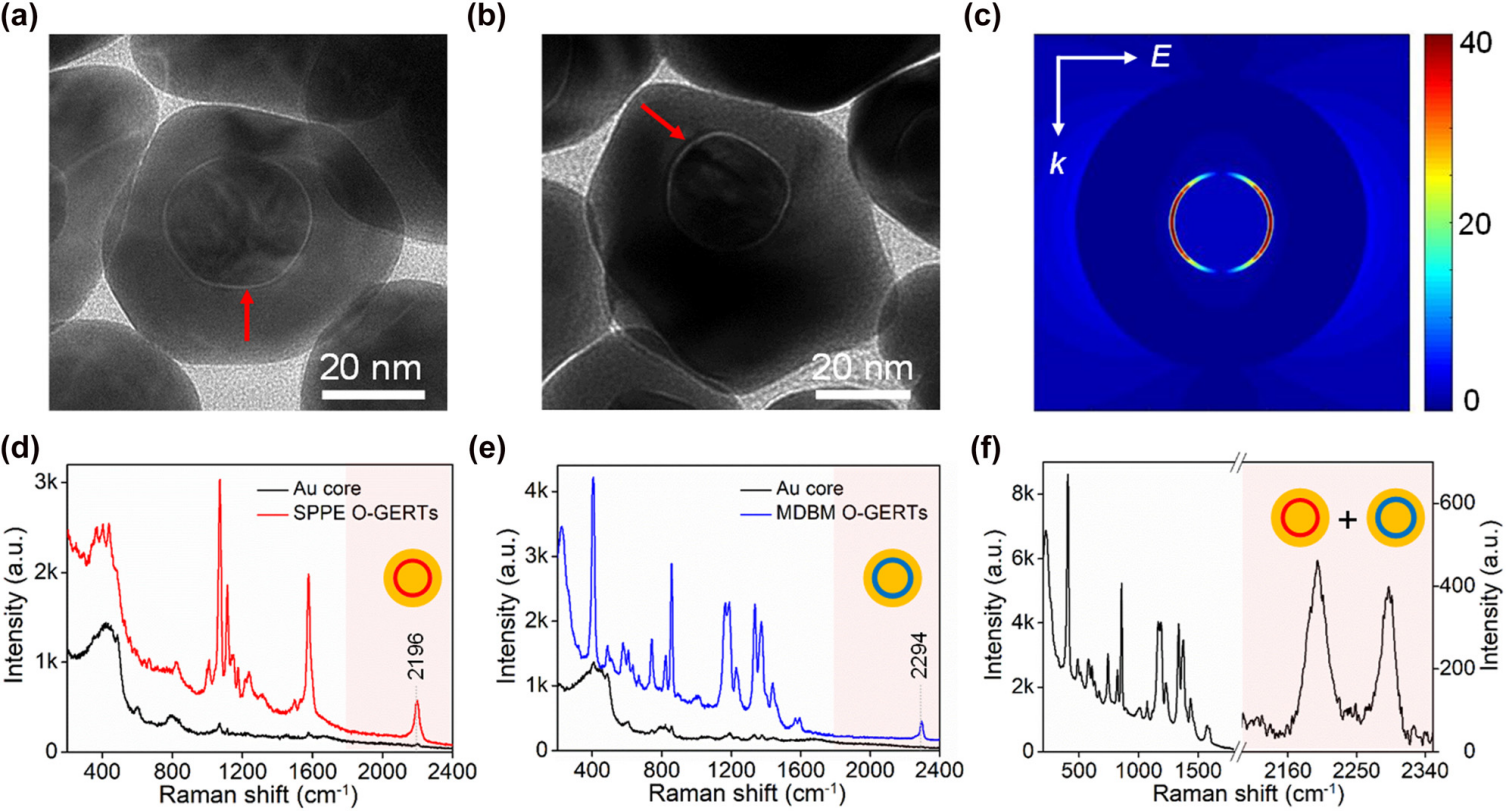
TEM images of (a) SPPE O-GERTs and (b) MDBM O-GERTs. Red arrows indicate the interior nanogaps of O-GERTs. (c) FDTD calculated electric field enhancement of a single O-GERT at 785 nm. SERS spectra of (d) SPPE-modified cores and SPPE O-GERTs, (e) MDBM-modified cores and MDBM O-GERTs, and (f) the mixture of both O-GERTs.

SPPE-embedded O-GERTs (SPPE O-GERTs) and MDBM-embedded O-GERTs (MDBM O-GERTs) are selected in following experiments due to superior sensitivity and sole band in the silent region (Figure S9). They were prepared with a NP size of 59 ± 5 and 61 ± 4 nm, respectively. High-resolution TEM images verify their core–shell structures spaced by a uniform gap with a thickness of approximately 0.9 and 0.8 nm, respectively (Figure 2a and b). They both show a single plasmon resonance peak in the visible range, red-shifted and broadened compared to the Au cores (Figure S10). The finite-difference time domain (FDTD) calculations indicate that electric field enhancement in the nanogap of a single GERT is obviously stronger than that of a single Au NP with a same size (Figures 2c and S11) [27, 28, 30]. This explains that both O-GERTs show signal increase in silent-region more than one order of magnitude compared to the corresponding Au NPs at off-resonance condition such as 785 nm (Figures 2d, e and S12). O-GERTs both present multiple Raman bands in the fingerprint region but a single one in the silent region (2196 cm^−1^ for SPPE O-GERTs and 2294 cm^−1^ for MDBM O-GERTs). This implies favorable spectral features for multiplexing. For example, a spectrum of the mixture of both O-GERTs with a 1:5 ratio shows that the fingerprint-region signal become largely overlapped, whereas the silent-region signals are completely separated (Figure 2f). Moreover, multiplexing of four types of O-GERTs shows resolvable silent-region signals, thus simplifying spectral analysis in detection and imaging (Figure S13). In addition, molecular engineering techniques allow further improvement of the multiplexing capability utilizing the orthogonality in the silent region [37, 38].

### 3.3 O-GERTs stability

**Figure 3:**
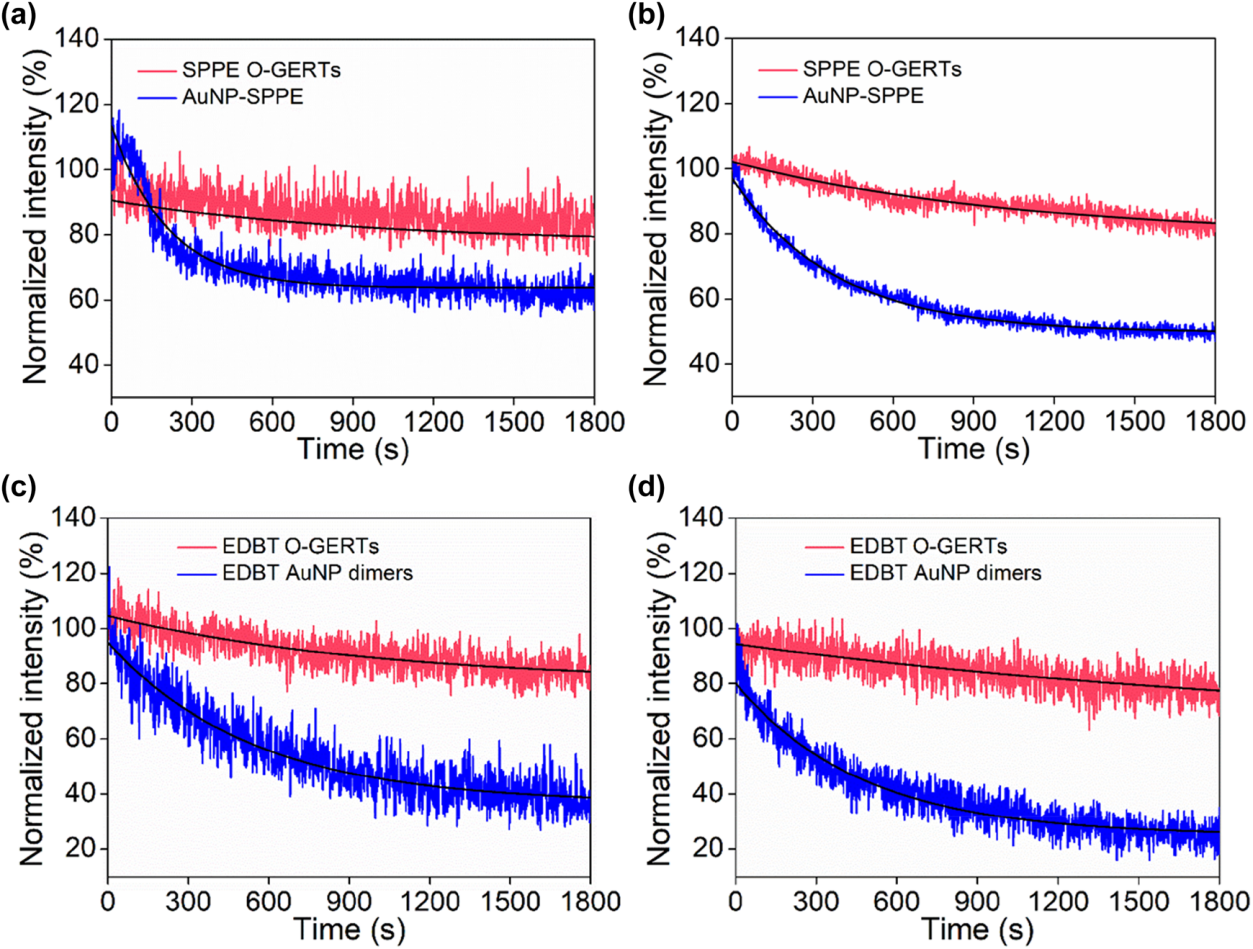
Photostability of SPPE O-GERTs and AuNP-SPPE tags under a laser power density of (a) 2.95 × 10^5^ and (b) 1.17 × 10^6^ W/cm^2^. Photostability of both tags were evaluated with integrated peak areas of Raman band at 2196 cm^−1^. Photostability of EDBT O-GERTs and EDBT AuNP dimers under the laser power density of (c) 2.95 × 10^5^ and (d) 1.17 × 10^6^ W/cm^2^. Photostability of both tags were evaluated with integrated peak areas of Raman band at 2205 cm^−1^. The photobleaching time constant is 542 s for EDBT AuNP dimers and 1008 s for EDBT O-GERTs under 2.95 × 10^5^ W/cm^2^, while the value is 473 and 2381 s under 1.17 × 10^6^ W/cm^2^, respectively.

We turned to evaluate the stability of O-GERTs. To highlight the effect of RR location on tag stability, we compared O-GERTs with Au NPs (60 nm in diameter) with RRs onto the surface. We first compared the photostability of SPPE O-GERTs and SPPE-modified Au NPs (AuNP-SPPE) by continuous laser irradiation for 30 min under different laser power densities: 2.95 × 10^5^ and 1.17 × 10^6^ W/cm^2^. Through quantitative analysis of photobleaching behaviors of time-resolved SERS trajectories [34, 35], it can be found that the photobleaching time constant is 210 s for AuNP-SPPE and 941 s for SPPE O-GERTs under 2.95 × 10^5^ W/cm^2^, while the value is 385 and 1063 s, respectively, under 1.17 × 10^6^ W/cm^2^ (Figure 3a and b). These results indicate that SPPE O-GERTs with a high photostability are more suitable for reduplicated bioimaging than AuNP-SPPE. The ultrahigh photostability of SPPE O-GERTs is mainly due to the reasons including: (1) the solid metallic shell well protects the built-in RRs to avoid possible desorption and minimizes photoinduced chemical reactions by isolation from the environment (oxygen, moisture, etc.); (2) the use of off-resonance laser excitation in the near-infrared (NIR) region (e.g., 785 nm) circumvents the plasmon resonance of GERTs in the visible region (e.g., 540 nm in Figure S10), which dramatically reduces the photoheating effects from the laser. In addition, the SPPE O-GERTs appear to be significantly more stable in common biological fluids including PBS, FBS, DMEM compared to AuNP-SPPE (Figure S14), due to minimized RR leakage or chemical damage from media. Similarly, MDBM O-GERTs show higher photo and biological stability compared to AuNP-MDBM tags (Figure S15). Moreover, both O-GERTs show long-term stability in water (Figure S16). It can be also found that O-GERTs exhibit superior stability than state-of-the-art nanostructures, including AuNP dimers and Au nanorods with absorption at 820 nm (Figures 3c, d, S17–20 and Table S1). These evaluations suggest that O-GERTs can work in different biological environments and conditions, indicating the notable advantages of embedding RRs in the intra-nanogaps of core–shell NPs.

### 3.4 Demonstration of signal orthogonality of O-GERTs against universal backgrounds

**Figure 4:**
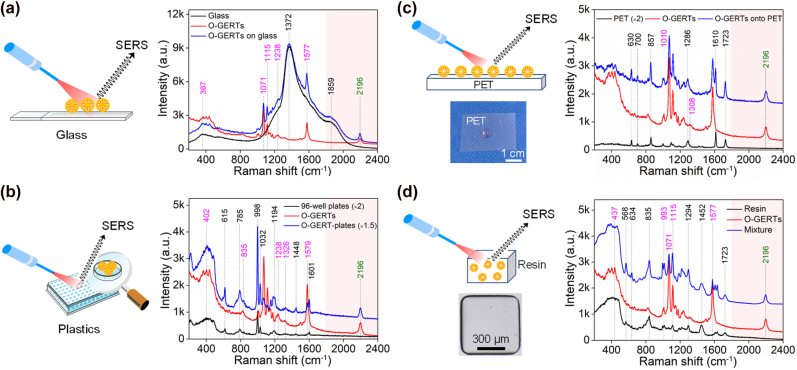
Schematic diagram of Raman measurements and the anti-interference capability of SPPE O-GERTs on different substrates: (a) glass, (b) plastics (96-well plates), (c) PET films and (d) resins. These peaks marked in black, magenta and green are from substrates, O-GERTs in the fingerprint region and O-GERTs in the silent region, respectively.

A good substrate for SERS measurements should have a low background to avoid masking the signals from the sample/tag. However, typical substrates exhibit non-negligible vibrations in the fingerprint region. SPPE O-GERTs were selected to investigate anti-interference properties against two common substrates (glass and 96-well plate). For glass, the broad band at 1372 cm^−1^, originated from photoluminescence [9, 39, 40], overlaps and modifies the lineshape of bands at 367, 1071, 1115, 1238 and 1577 cm^−1^ from O-GERTs deposited on glass (Figure 4a). By contrast, the signal at 2196 cm^−1^ from O-GERTs remains entirely unaffected. Similar anti-interference phenomena were observed when measuring O-GERTs on the 96-well plate made of polystyrene [41] (Figure S21 and Table S2): intact orthogonal signal at 2196 cm^−1^ of O-GERTs but severely interfered signals in the fingerprint region (Figure 4b). These examples indicate that the orthogonal signal of SPPE O-GERTs is interference-free against these common substrates widely used in analytical research and clinical diagnosis. We also studied anti-interference properties of SPPE O-GERTs against polymer film (e.g., polyethylene terephthalate, PET) and resin (Figures S22, S23 and Tables S3, S4), which are favorable substrates and media for information encoding when combined with SERS tags [42], [43], [44]. The SPPE O-GERTs dropped on the surface of PET film show that the bands at 630, 700, 857, 1286, 1610 and 1723 cm^−1^ are interfered from the PET background and the lineshape of the bands at 1010 and 1308 cm^−1^ is modified (Figure 4c). For resin embedded with O-GERTs and shaped into a square micropattern by SlipChip-based microfluidic technique [36], the fingerprint-region signals of O-GERTs are influenced upon resin incorporation (Figure 4d). Careful examinations show that even the strong peak at 1071, 1115 and 1577 cm^−1^ from SPPE O-GERTs can be seriously affected by resins. By contrast, the signal at 2196 cm^−1^ of O-GERTs in the presence of both polymers remains unaffected.

**Figure 5:**
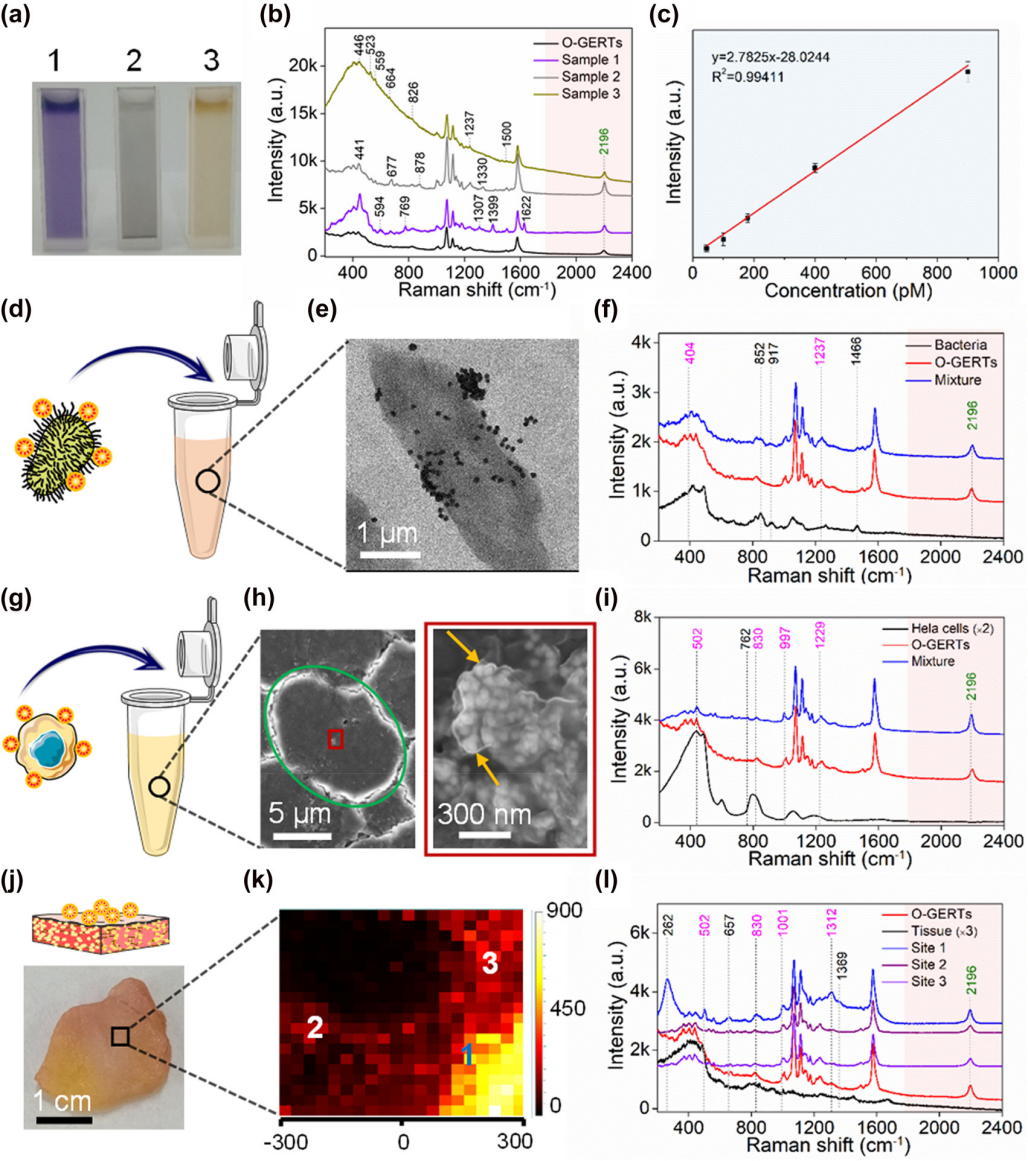
Anti-interference of O-GERTs in detection and imaging. (a) Photographs of three samples of waste water and (b) the anti-interference evaluation of SPPE O-GERTs in these samples. (c) Calibration curves created by plotting the peak at 2196 cm^−1^ with various concentrations of SPPE O-GERTs in sample 1. (d and e) Schematic diagram and TEM image of mixture of CB bacteria and SPPE O-GERTs. The anti-interference capability of SPPE O-GERTs in (f) CB bacteria, (i) HeLa cells and (l) tissue with three sites. (g and h) Schematic diagram and SEM images of mixture of HeLa cells and SPPE O-GERTs. The green circle outlines a cell and yellow arrows indicate the O-GERTs. (j) Schematic diagram and photograph of SPPE O-GERTs onto the breast cancer tissue. (k) Raman mapping of the tissue with an area of 600 × 500 μm^2^ and a resolution of 20 × 20 pixels.

SERS tags are often used as labels for *in vitro* detection [21, 32, 45, 46]. In this regard, three samples of waste water from different sources (Figures 5a and S24) are chosen to demonstrate anti-interference properties of O-GERTs in liquid media. By incubating SPPE O-GERTs with waste liquid, the corresponding Raman spectra for all samples show that many peaks distinct from the O-GERTs appear in the fingerprint region, which are likely to be derived from various molecules in sewage that possess multiple vibrations [31, 32]. By comparison, the signal at 2196 cm^−1^ remains unchanged (Figure 5b). As an example, an excellent linear relationship between concentration and intensity at 2196 cm^−1^ in sample 1 is well-established, while the intensity at 1577 cm^−1^ band does not have a good linear relationship with the concentration (Figures 5c and S25), suggesting that even the Raman intensity of the strongest peak at 1071 cm^−1^ from SPPE O-GERTs can be slightly affected by the backgrounds, and the silent-region signal of the O-GERTs can achieve better quantitative analysis than the fingerprint-region signal of the O-GERTs in complex environments.

SERS tags are also widely used as sensing and imaging probes in biological samples [47], [48], [49]. We first investigated signal orthogonality of SPPE O-GERTs against clostridium bolteae (CB) bacteria (Figure S26 and Table S5). For sample preparation, we mixed SPPE O-GERTs with CB bacteria suspension (Figure 5d), as evidenced from the TEM image in Figure 5e. Spectral analysis shows that the bands at 852, 917 and 1463 cm^−1^, which derive from biomolecules such as proteins, DNA and amides, appear in O-GERT-bacterium mixed spectra (Figure 5f) [48]. Moreover, the lineshape of the peaks at 404 and 1238 cm^−1^ changes because of the influence of nearby peaks at 416 and 1266 cm^−1^, which are assigned to carbohydrate C–C–C bending and amide III vibration in CB bacteria (Figure 5f) [50, 51]. Similar interference phenomena were also observed in the mixture of SPPE O-GERTs and Hela cells (Figures 5g–i, S27 and Table S6), consistent with previous reports [12, 22]. By contrast, the Raman band at 2196 cm^−1^ remains unaffected in combination with CB bacteria and Hela cells. Moreover, the Raman mapping of SPPE O-GERTs attached onto the surface of breast cancer tissue on an area of 600 × 500 μm^2^ (Figures 5j, k, S28 and Table S7) indicates that the signals in the fingerprint region of O-GERT are to some extent influenced by tissue background at different sites, while the signal at 2196 cm^−1^ is nonimpacted (Figure 5l). In short, these examples reveal that the orthogonal signal of O-GERTs is interference-free against common biological backgrounds, offering potential for accurate evaluation in biosensing and bioimaging.

### 3.5 Applications of O-GERTs for information security

**Figure 6:**
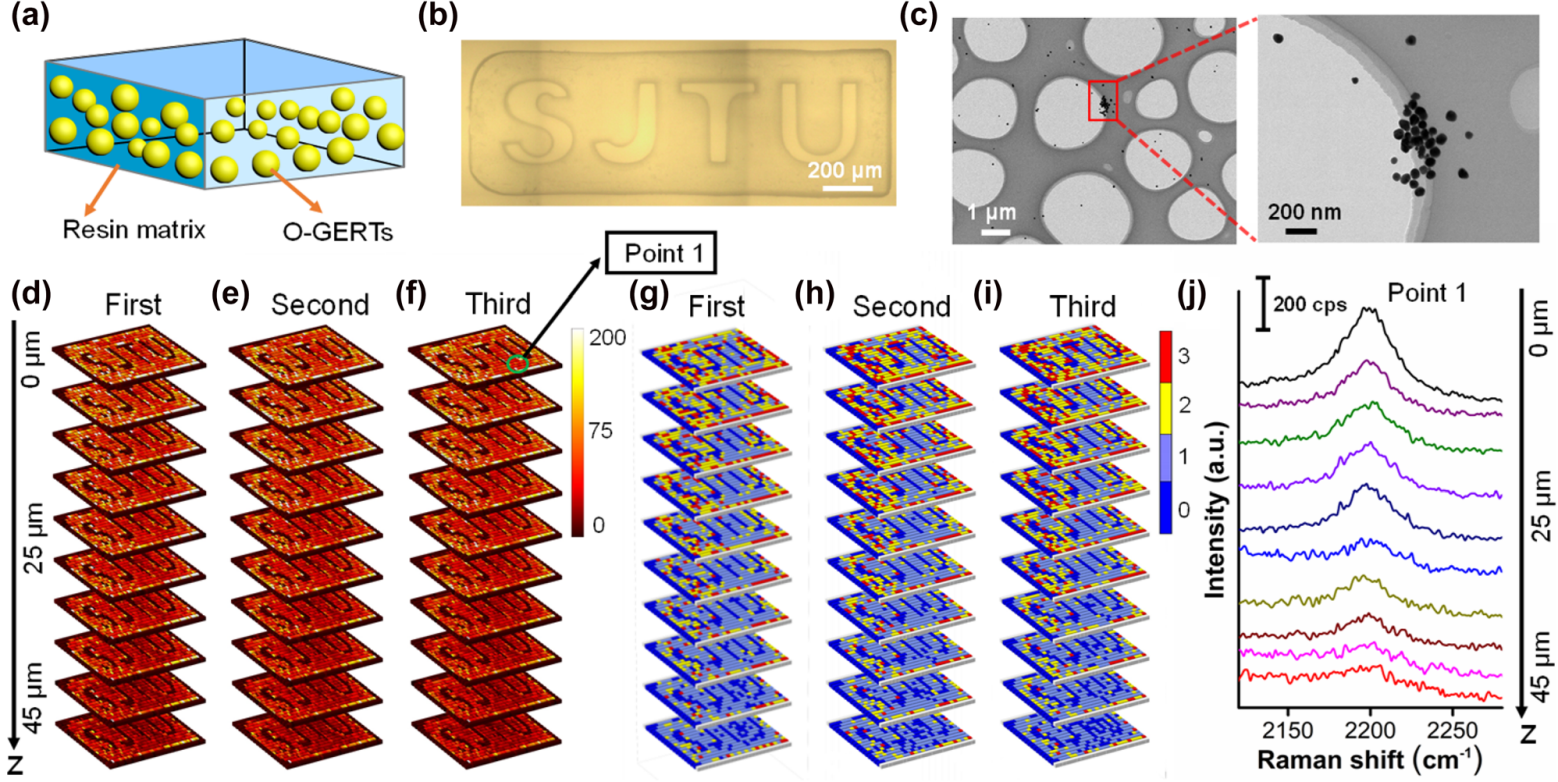
Security labels using SPPE O-GERTs embedded in resins. (a) Schematic diagram of resins with embedded SPPE O-GERTs. (b) Representative bright-field image of a security label with a pattern of “SJTU”. (c) Typical TEM images of ultrathin sections from the security label, showing the random distribution of embedded SPPE O-GERTs. (d–f) 3D Raman images from three repeated readouts acquired at ten focal planes of *z* = 0, 5, 10, 15, 20, 25, 30, 35, 40 and 45 μm, respectively, and (g–i) the corresponding digitized quaternary codes of the labels. (j) SERS spectra of point 1 obtained at various *z*-focal planes, as indicated in panel (f). Raman images, digitized codes and intensity profiles are derived using the orthogonal band (2196 cm^−1^) of SPPE O-GERTs.

Inspired by the ultrastable interference-free signal of O-GERTs, we applied them to design a 3D physically unclonable security label for anticounterfeiting and to overcome the typical overlapped background signals from the polymer protection layer. A Raman-based security label usually involves the process of manufacture, readout, digitization and authentication [42, 44, 52]. We first started to manufacture the label by dispersed SPPE O-GERTs in the resin matrix (Figure 6a), which plays extremely important roles as a rigid scaffold for the formation of 3D label and as a protection layer to avoid the physical damage of labels in use. The security label was then molded into a rectangle with a thickness of 50 μm by the SlipChip microfluidic technique, which has a hollow “SJTU” micropattern corresponding to the acronym of “Shanghai Jiao Tong University” for user recognition (Figure 6b). Representative TEM images from ultrathin sections of the label in a thickness of 150 nm reveal that the O-GERTs are randomly distributed in the resin (Figure 6c). This disorder perfectly meets the requirement of unclonable feature for anticounterfeiting labels. Through layer-by-layer scanning, we read this 3D label by performing the Raman imaging of ten layers with a resolution of 52 × 19 pixels per layer at different focal planes along *z*-axis. The afore-mentioned anti-interference of SPPE O-GERTs against the resin (Figure 4d) allows to provide sufficient signal-to-noise ratio in the readout process, and clearly reveal the “SJTU” pattern using the orthogonal band at 2196 cm^−1^ in each layer totally across ten layers (*z* = 0, 5, 10, 15, 20, 25, 30, 35, 40 and 45 μm, respectively) in three repeated measurements (Figure 6d–f). Then, the readout signals were digitized based on the Raman intensity levels (2196 cm^−1^) at each pixel with a quaternary encoding method. The digitized codes for the label show intrinsically distinct distributions of intensity levels between different layers (Figure 6g). Moreover, the ultrahigh photostability of the O-GERTs guarantees a similarity index of more than 97% within three repeated measurements (Figure 6h and i), favorable for repeated readouts during practical uses. The Raman spectra at point 1 (Figure 6f) across different ten layers (with different *z* values) show that the Raman intensity at 2196 cm^−1^ can be distinguished from the background signal of resins (Figure 6j). These *z* values in the labels employed as encryption layers are unknown to the public and only authorized individuals, who know the correct decoding rule of the O-GERTs in 3D spaces, are able to access the secret information, thus significantly improving the security level of the anticounterfeiting label. To our knowledge, it is the first time 3D unclonable security label has been reported. Compared to conventional 2D Raman anticounterfeiting platforms reported in our recent work and in other groups [44, 53], [54], [55], [56], this 3D encoding platform not only enables up to 10-fold denser data storage along the *z*-axis using only a single reporter molecule within the same 2D area.

To preliminarily authenticate these PUF labels, we manufactured a number of circular PUF labels with well-defined sizes (900 μm in diameter and 50 μm in thickness) by our SlipChip method (Figure S29a) and randomly selected three labels to read and authenticate. The readout of each label was repeated three times. Raman mapping from three measurements of the same label show very similar intensity distribution attributed to ultrahigh photostability of SPPE OGERTs, while these different ones display vastly different (Figure S29b). Based on intensity levels at each pixel in the Raman mapping, digitized codes of these three labels show different digital distribution while the same label for three repeated measurements are highly similar (Figure S29c). The calculated similarity index shows that the same label clearly show *I* values higher than 96%, while the three different ones show values below 58% (Figure S29d). These results suggest that it is possible to distinguish real labels from the duplicate ones. In the actual product authentication process, PUF labels are pasted onto products such as drugs, read by a Raman spectrometer, digitized by software, and stored in database. During production circulation, the PUF labels will be read and digitized at each stage (including distribution, retail and the end user) to from digital secret keys. If a digital key obtained from a user match in the database, the product be verified as **AUTHENTIC**; otherwise it will be suspected as **COUNTERFEIT**. Coupled with interference-free readouts, excellent signal reproducibility, and remarkable encoding capacity, our security label represents a new anticounterfeit technique with the potential to be commercially implemented for real-world forensic authentications.

## 4 Conclusions

In conclusion, we have designed and synthesized a series of O-GERTs, and we identified two ultrastable O-GERTs embedded with orthogonal RRs for interference-free SERS studies. We demonstrate that the silent-region signals of O-GERTs are anti-interference against various backgrounds including glass, polymer, bacteria, cells and tissues, thus highlighting O-GERTs as universal optical tags for accurate and reliable detection. Moreover, these O-GERTs show much higher photo and biological stability compared to conventional SERS tags with RRs on the surface and Au NP dimers with RRs in the inter-nanogaps. This proof-of-concept study not only helps understand the fundamental concepts of orthogonal SERS tags but also advances their wide potential applications such as the 3D unclonable security label.

## Supplementary Material

Supplementary Material Details
