# Supplementary material for: Orthogonal gap-enhanced Raman tags for interference-free and ultrastable surface-enhanced Raman scattering
Source: Nanophotonics. 2022 Mar 28;11(8):1549–60. doi: 10.1515/nanoph-2021-0689 (PMC11501518; doi:10.1515/nanoph-2021-0689)
Supplement: Supplementary file 1 — Supplementary Material Details [file j_nanoph-2021-0689_suppl.docx]

**Supporting Information**

**Orthogonal gap-enhanced Raman tags for interference-free and ultrastable surface-enhanced Raman scattering**

Jin Li ^a^, Fugang Liu ^a^, Chang He ^a^, Feng Shen ^a^, and Jian Ye ^a,b,c,^*

^a^ State Key Laboratory of Oncogenes and Related Genes, School of Biomedical Engineering, Shanghai Jiao Tong University, Shanghai, 200030, P. R. China

^b^ Shanghai Key Laboratory of Gynecologic Oncology, Ren Ji Hospital, School of Medicine, Shanghai Jiao Tong University, Shanghai, 200127, P. R. China

^c^ Institute of Medical Robotics, Shanghai Jiao Tong University, Shanghai 200240, P. R. China

*Corresponding author: Jian Ye (E-mail address: [yejian78@sjtu.edu.cn](mailto:yejian78@sjtu.edu.cn))

Table of contents

# 1. Synthetic chemistry

**2. Supplementary Figures S1-29**

**3. References**

# Synthetic chemistry

# General information.

All syntheses were carried out under an inert atmosphere (nitrogen or argon) using the standard Schlenk technique unless otherwise stated. Solvents were distilled under nitrogen from sodium/benzophenone (hexane and diethyl ether) or calcium hydride (dichloromethane) prior to use. 4-(2,5-Dibromophenyl)pyridine was purchased from Wuhan Greatwall Biochemical Co., Ltd.. S-(4-ethynylphenyl) ethanethioate (SEE), S-(4-((trimethylsilyl)ethynyl)phenyl) ethanethioate (STE), (4-ethynylphenyl)(methyl)sulfane (EMS) and trimethyl((4-(methylthio)phenyl)ethynyl)silane (TMPS) were synthesized according to our previous work [1]. The other reagents and solvents were purchased from Sigma-Aldrich and used without further purification. NMR spectra was collected on a Brucker AV-500 spectrometer. ^1^H and ^13^C NMR chemical shifts (δ) are relative to tetramethylsilane. The high-resolution mass spectra (HRMS) experiments were performed on a Bruker En Apex Ultra 7.0T FT-MS.

**Synthesis of compound 1.** Compound 1 (see below Scheme S1) was synthesized according to the literature [1, 2]. A mixture of 4-(2,5-dibromophenyl)pyridine (908.62 mg, 2.903 mmol), CuI (22.09 mg, 0.116 mmol), and Pd(PPh_3_)_2_Cl_2_ (81.42 mg, 0.116 mmol) in a 100 mL three-neck flask was degassed and refilled with nitrogen. After repeating this procedure three times, THF/Hunig’s base solvent mixture (10 mL: 10 mL) and phenylacetylene (296.50 g, 2.903 mmol) were added by syringe. The reaction solution was stirred at 50 ^o^C for 12-48 h, then the solvent was evaporated under reduced pressure and the remaining residue was further purified by column chromatography on silica to afford compound 1 (456 mg, 47%) as a colorless solid.

**Synthesis of S-(4-((4-(phenylethynyl)-2-(pyridin-4-yl)phenyl)ethynyl)phenyl) ethanethioate (SPPE).** SPPE was synthesize by a reported method [3]. As sketched in Scheme S1, SEE (82 mg, 0.465 mM), Pd(PPh_3_)_2_Cl_2_ (10.95 mg, 0.0156 mmol), CuI (29.71 mg, 0.0156 mmol) and compound 1 (130 mg, 0.390 mmol) were added to a Schlenk flask containing THF/Hunig’s base solvent mixture (8 mL: 8 mL). The reaction mixture was deoxygenated by performing three freeze pump-thaw cycles, after which the flask was backfilled with nitrogen and sealed. The mixture was then stirred for 12-48 h at 55 °C until the reaction was completed verified by thin-layer chromatography. The volatiles were then removed in vacuo and the solids were further purified by column chromatography on silica gel to provide 124 mg of white solid product in 74% yield.

**Synthesis of compound 2.** Thioacetic acid S-(4-iodo-phenyl) ester (834 mg, 3 mmol), Pd(PPh_3_)_4_ (5 mol%, 173 mg, 0.15 mmol) and CuI (5 mol%, 29 mg, 0.15 mmol) were added in a dried flask and dissolved in a mixture solution of well-degassed *i*-Pr_2_NEt (4 mL) and THF (16 mL). After stirring for 5 min, S-(4-ethynylphenyl) ethanethioate (530 mg, 3 mmol) in THF (10 mL) was added dropwise within 1 h under an argon atmosphere, the reaction mixture was stirred at 50 ^o^C for 24-48 h until the reaction was finished evidenced by TLC. The mixture was poured into water and the aqueous layer was extracted with CH_2_Cl_2_ (3 × 30 mL), the combined organic layers were washed with saturated brine (3 × 20 mL) and dried over MgSO_4_. The solvents were removed by vacuum evaporation to afford a dark yellow crude product. After that, the crude product was purified by column chromatography on silica gel to provide 424 mg of white solid product in 87% yield.

**Synthesis of 4,4'-(ethyne-1,2-diyl)dibenzenethiol (EDBT)**. EDBT was synthesized according to our works [1, 4]. Compound 2 (326 mg, 1 mmol) was dissolved in chloroform-methanol mixture (5 mL: 15 mL) and 2.5 M HCl solution in methanol (20 mL) was added. The mixture was refluxed in nitrogen atmosphere for 5 h. After that, the reaction mixture was concentrated in vacuum and the crude product was purified by column chromatography on silica gel to provide 189 mg of yellow solid product in 78% yield.





**Scheme S1.** Synthesis of SPPE and EDBT reporters. Reagents and conditions: (i) Pd(PPh_3_)_2_Cl_2_, CuI, 24-48h, 50 °C. (ii) Pd(PPh_3_)_2_Cl_2_, CuI, 55 °C, 48 h. (iii) Pd(PPh_3_)_4_, CuI, 24-48 h, 50 °C; (iv) HCl-MeOH, CHCl_3_, 5 h, rt.

**Characterization of SPPE**: ^1^H NMR (400 MHz, DMSO-d6) δ (ppm): 8.73 (s, 2H), 7.38-7.81 (m, 14 H), 2.45 (s, 3H). ^13^C NMR (700 MHz, DMSO-d6) δ (ppm) = 192.78, 149.55, 145.96, 134.47, 133.34, 131.95, 131.65, 131.42, 129.15, 128.76, 123.79, 123.29, 122.74, 121.70, 120.19, 93.47, 91.95, 89.47, 88.42, 30.22. HRMS (ESI): m/z calcd for [C_29_H_29_NOS+H], 430.1266; found: m/z 430.12527: [M+H]^+^.

**Characterization of EDBT**: ^1^H NMR: (600 MHz, CDCl_3_) δ 7.37 (d, J= 6.00 Hz, 4H), 7.24 (d, J= 12.00 Hz, 4H), 3.51 (s, 2H). ^13^C NMR (600 MHz, CDCl_3_) δ (ppm) = 132.23, 131.96, 129.09, 120.48, 89.41. HRMS (MALDI): m/z calcd for C_14_H_10_S_2_: 242.0224, found: 242.02184: M^+^.

**Supplementary Figures S1-29**


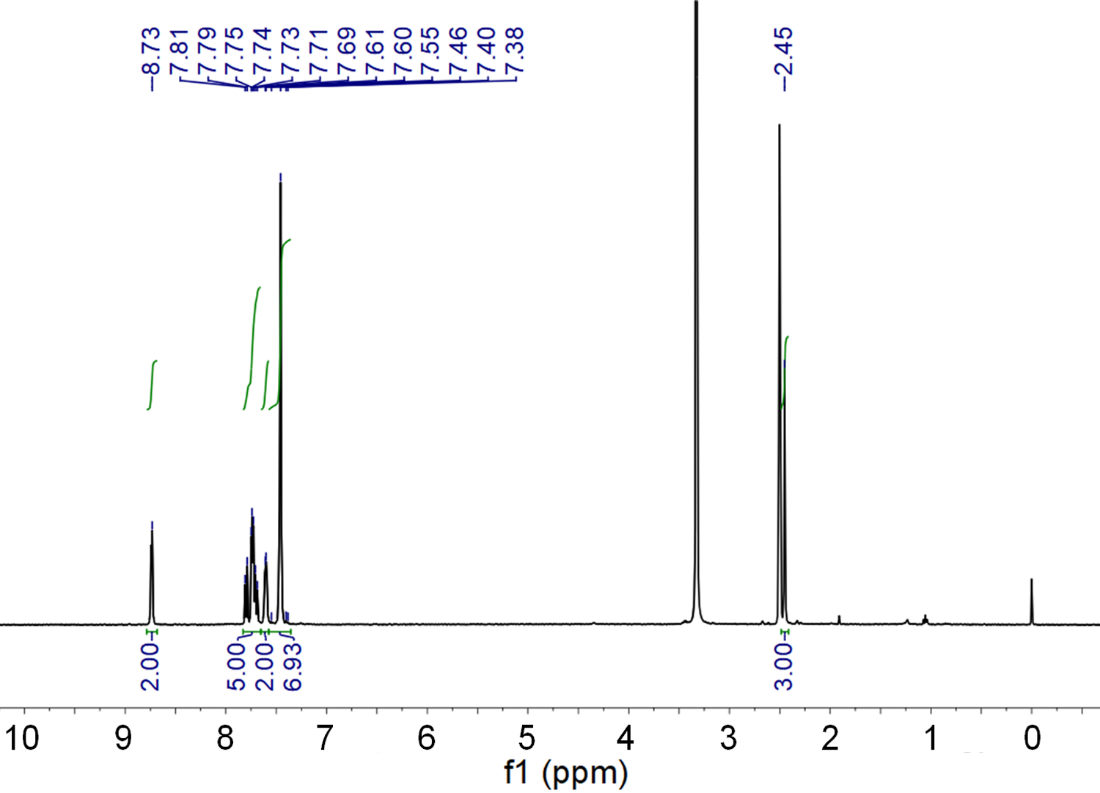


**Figure S1.** The ^1^H NMR (400 MHz, DMSO-d6) spectrum of compound SPPE.


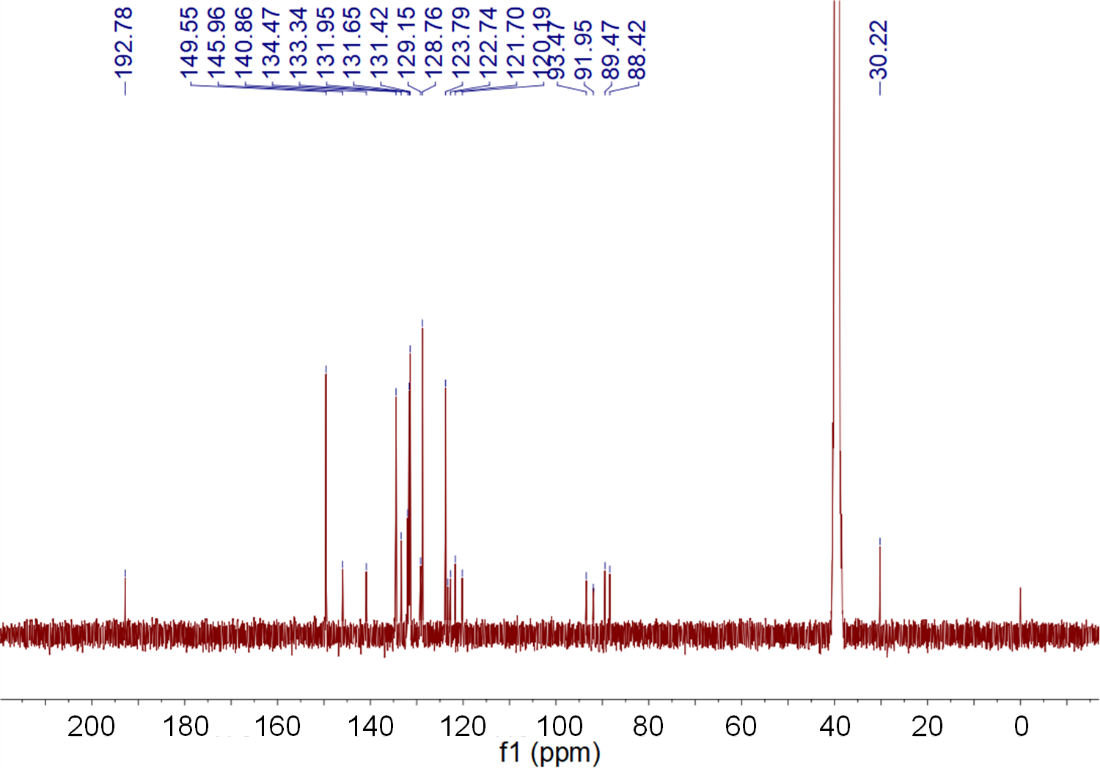


**Figure S2.** The ^13^C NMR (700 MHz, DMSO-d6) spectrum for compound SPPE.

**Figure S3.** The ESI-MS spectrum of SPPE. Calculated for [C_29_H_29_NOS+H] = 430.1266, ESI, found: m/z 430.12527: [M+H]^+^.


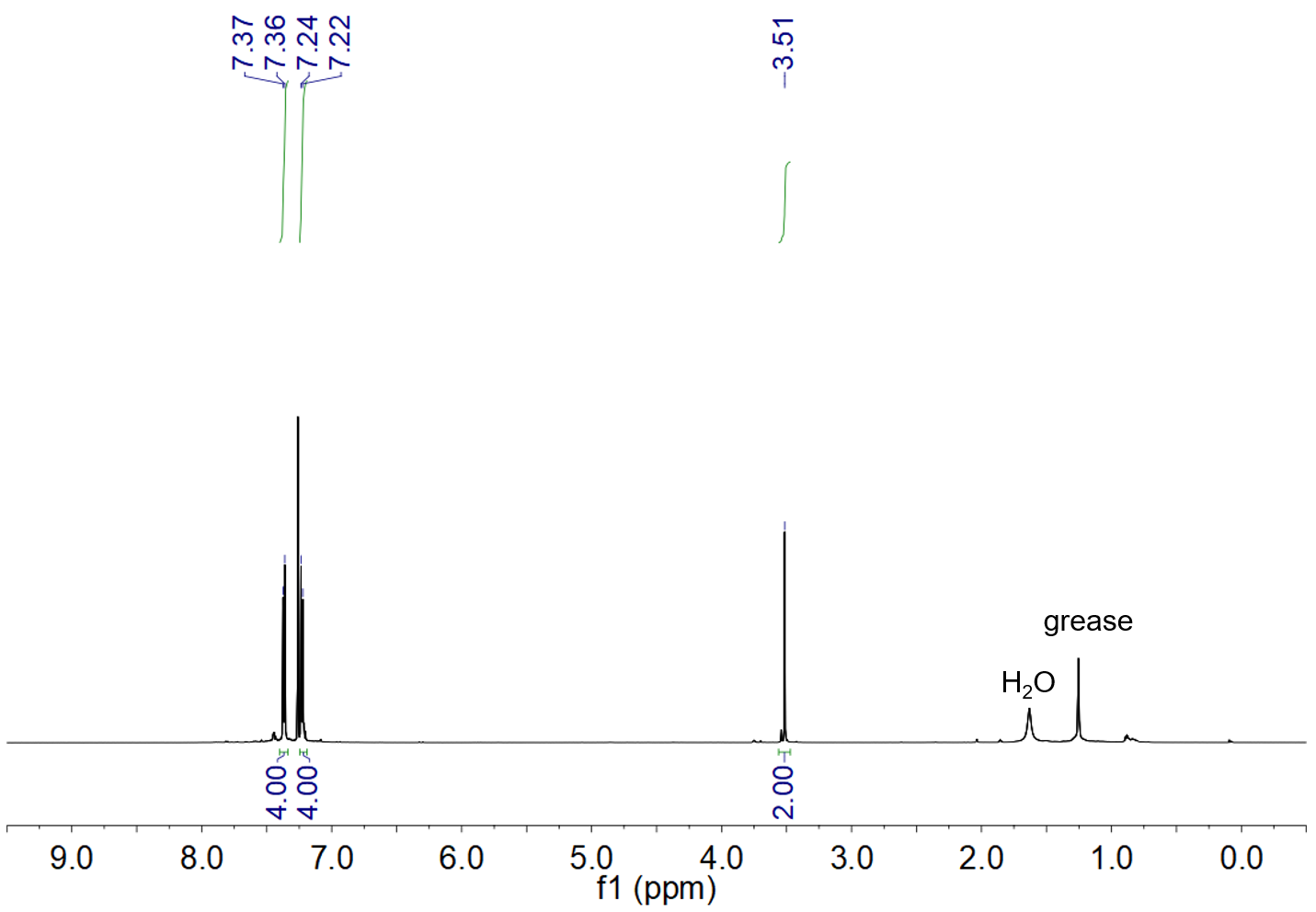


**Figure S4.** The ^1^H NMR (600 MHz, CDCl_3_) spectrum of compound EDBT.


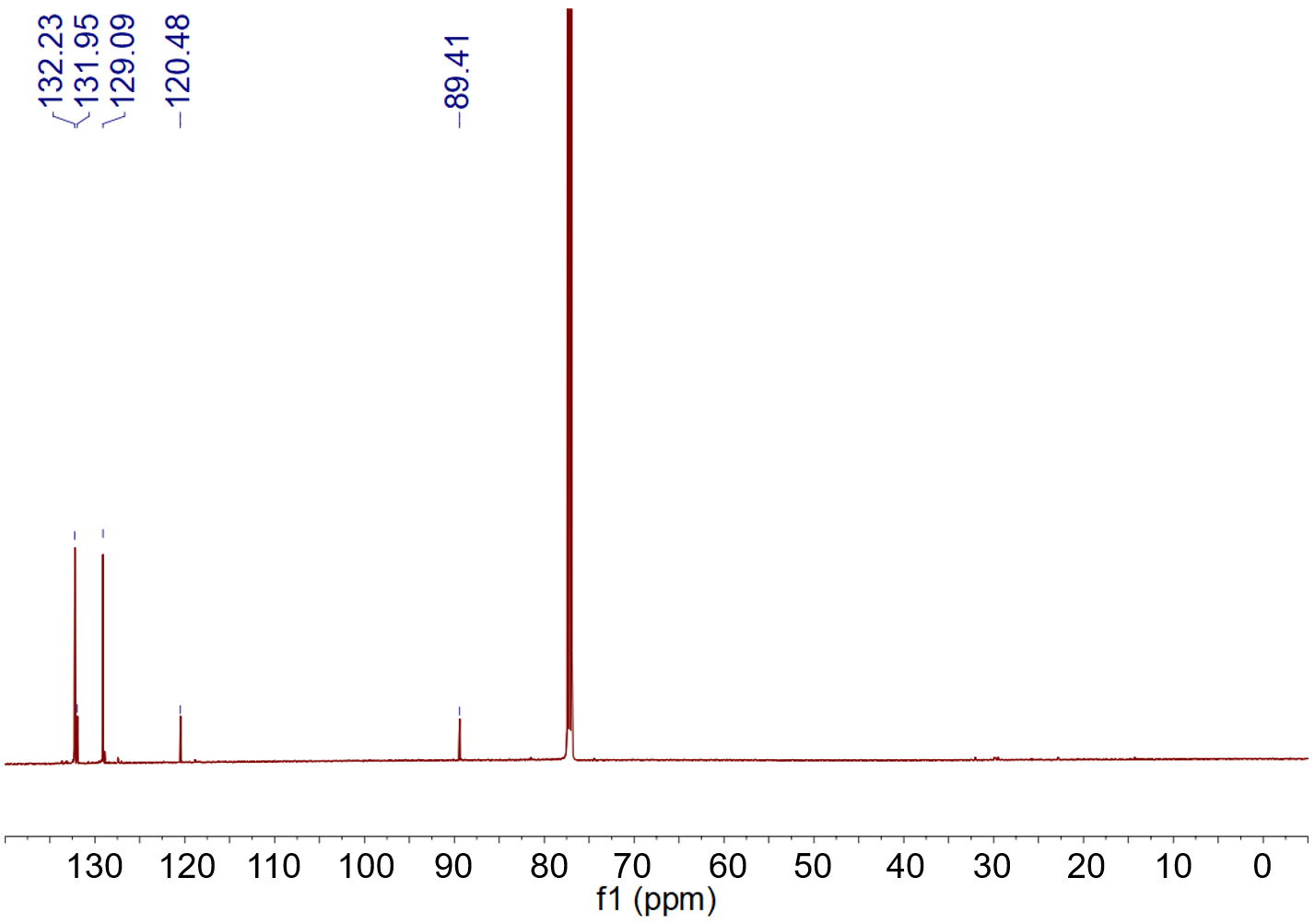


**Figure S5**. The ^13^C NMR (600 MHz, CDCl_3_) spectrum of compound EDBT.


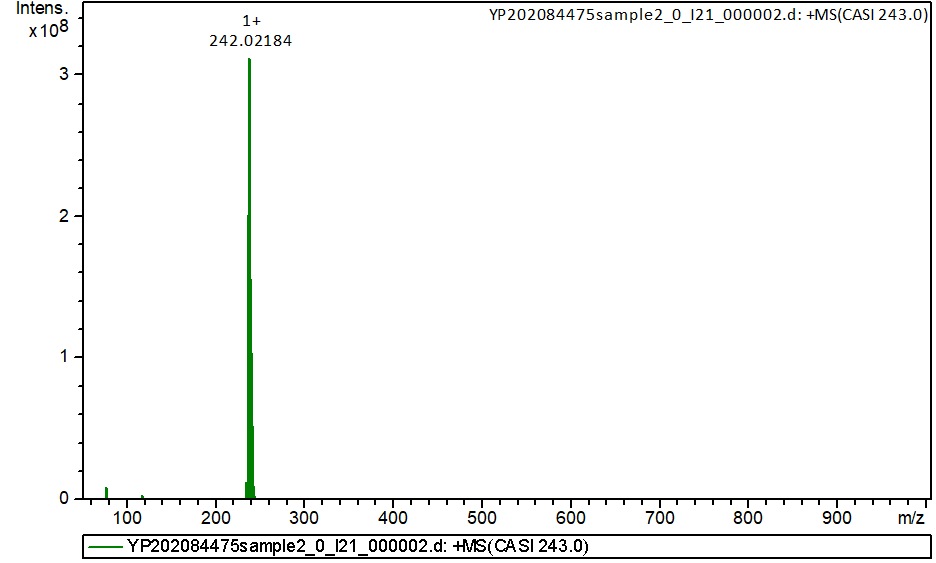


**Figure S6.** The MALDI-MS spectrum of EDBT. Calculated for [C_14_H_10_S_2_] = 242.0224, found: m/z 242.02184: M^+^.


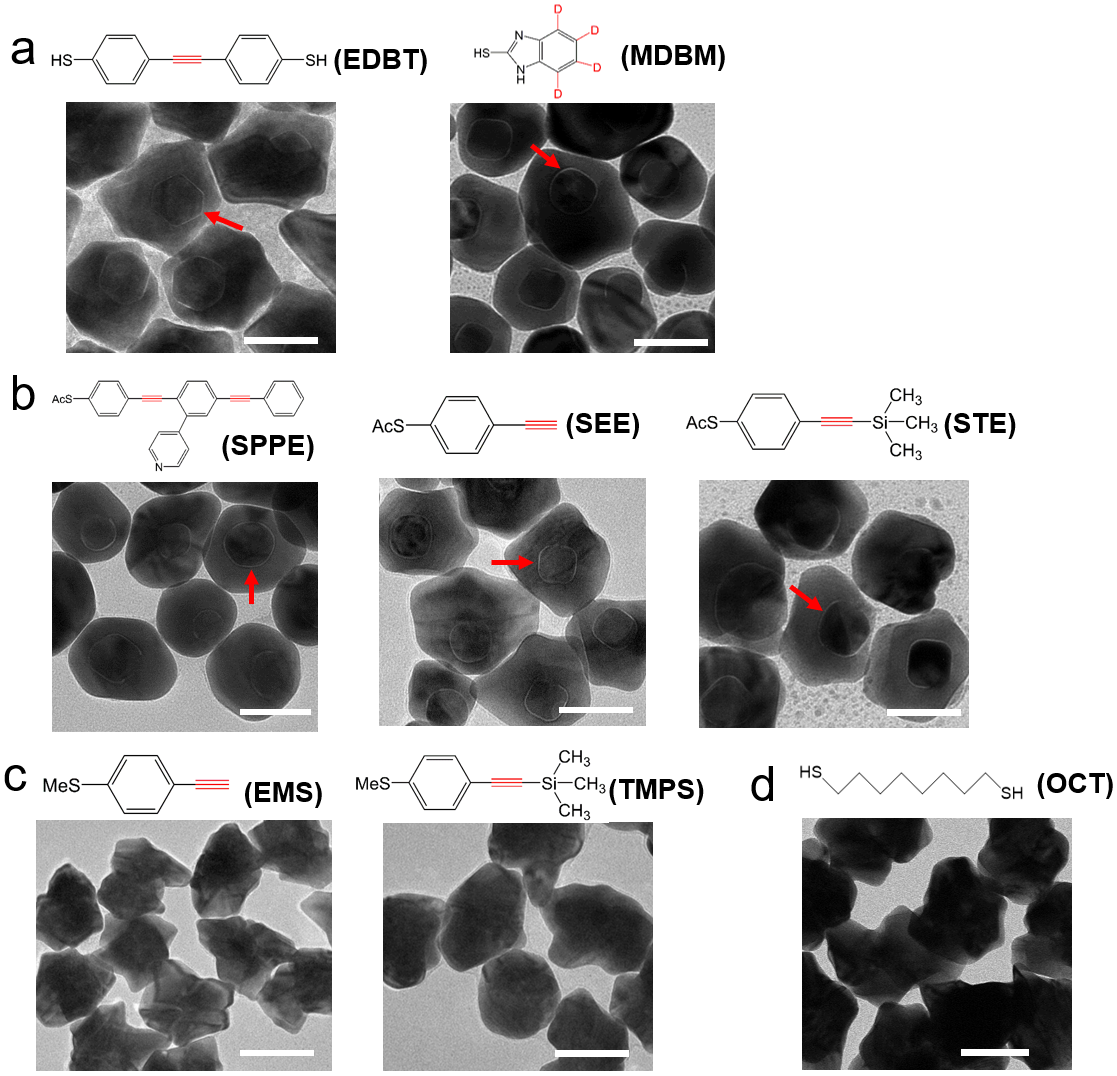


**Figure S7.** TEM images of O-GERTs with embbed Raman reproters with (a) sulfhydryl group, (b) acetyl sulfide group, (c) methyl sulfide group, and (d) a flexible backbone chain, incluging 4,4'-(Ethyne-1,2-diyl)dibenzenethiol (EDBT), 2-mercapto-4,5,6,7-d4-benzimidazole (MDBM), s-(4-((4-(phenylethynyl)-2-(pyridin-4-yl)phenyl)ethynyl)-phenyl) ethanethioate (SPPE), S-(4-ethynylphenyl) ethanethioate (SEE), s-(4-((trimethylsilyl)ethynyl)phenyl) ethanethioate (SPE), (4-ethynylphenyl)(methyl)sulfane (EMS), trimethyl((4-(methylthio)phenyl)ethynyl)silane (TMPS), and 1,8-octanedithiol (OCT). Red arrows indicate the internal nanogaps of O-GERTs. All scale bars are 50 nm. Note that sulfydryl and acetyl sulfide groups have a strong affinity to gold, while the binding mechanism is controversial and needs further study [5, 6].


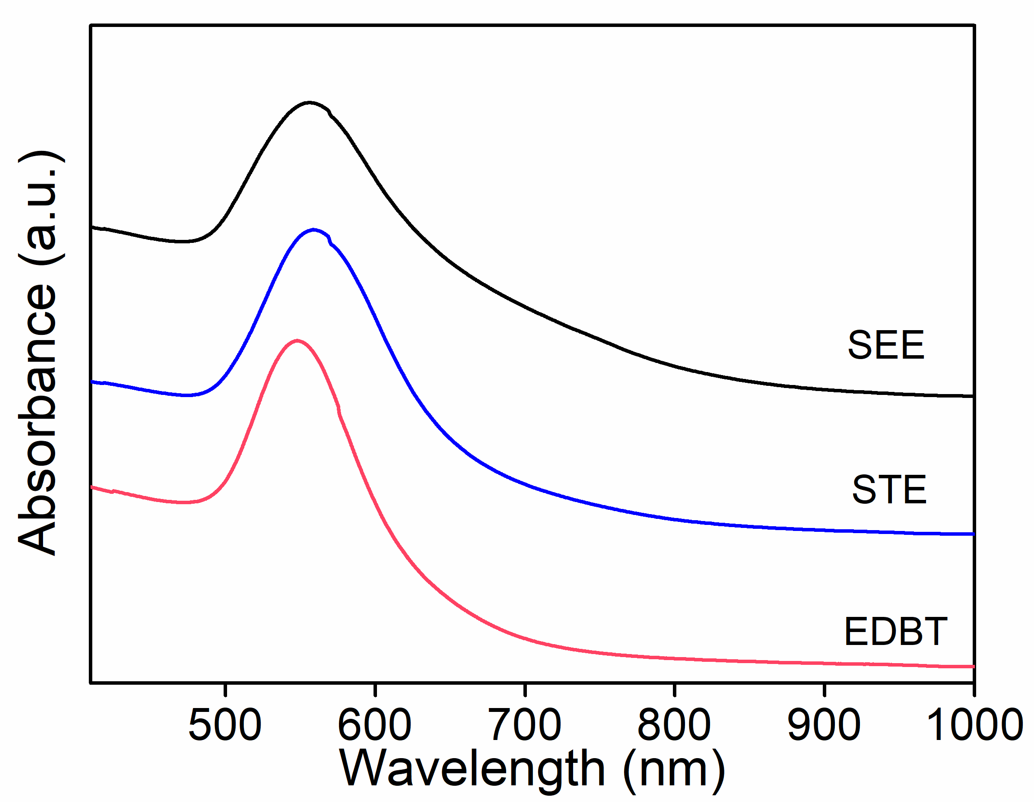


**Figure S8**. UV-Vis spectra of O-GERTs with embedded Raman reporters SEE, SPE and EDBT.





**Figure S9.** Raman spectra of seven NPs (with the same concentration) fabricated from a wet-chemistry approach using Raman reporters of EDBT, SEE, SPE, SPPE, MDBM, EMS, and TMPS, respectively. The molecules with methylthio group (EMS and TMPS) are not able to form uniform intra-nanogaps and show weak signals in the silent region. SPPE O-GERTs exhibit the highest Raman intensity in the silent region. MDBM O-GERTs display single Raman band (2294 cm^-1^) in the silent region and can be clearly distinguished from SPPE O-GERTs (2196 cm^-1^).


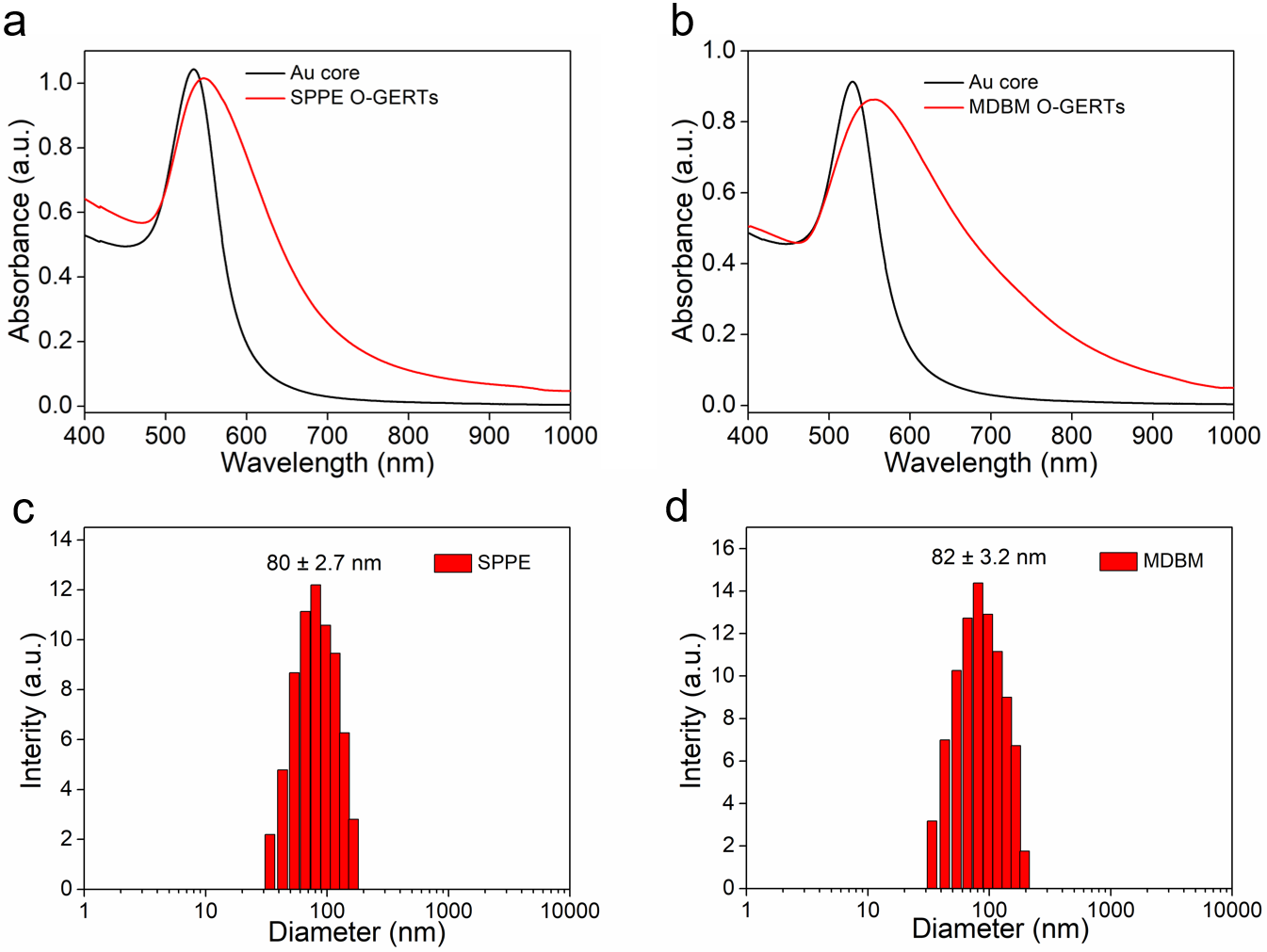


**Figure S10**. (a) UV-Vis spectra of Au cores chemisorbed with SPPE (black) and SPPE O-GERTs (red). (b) UV-Vis spectra of Au cores chemisorbed with MDBM (black) and MDBM O-GERTs (red). The insets in (a, b) show the typical ruby color of both aqueous O-GERTs, respectively. After formation of O-GERTs from Au cores, the resonance peaks for both O-GERTs show redshift and broadening. (c-d) Hydrodynamic diameters of SPPE O-GERTs and MDBM O-GERTs.


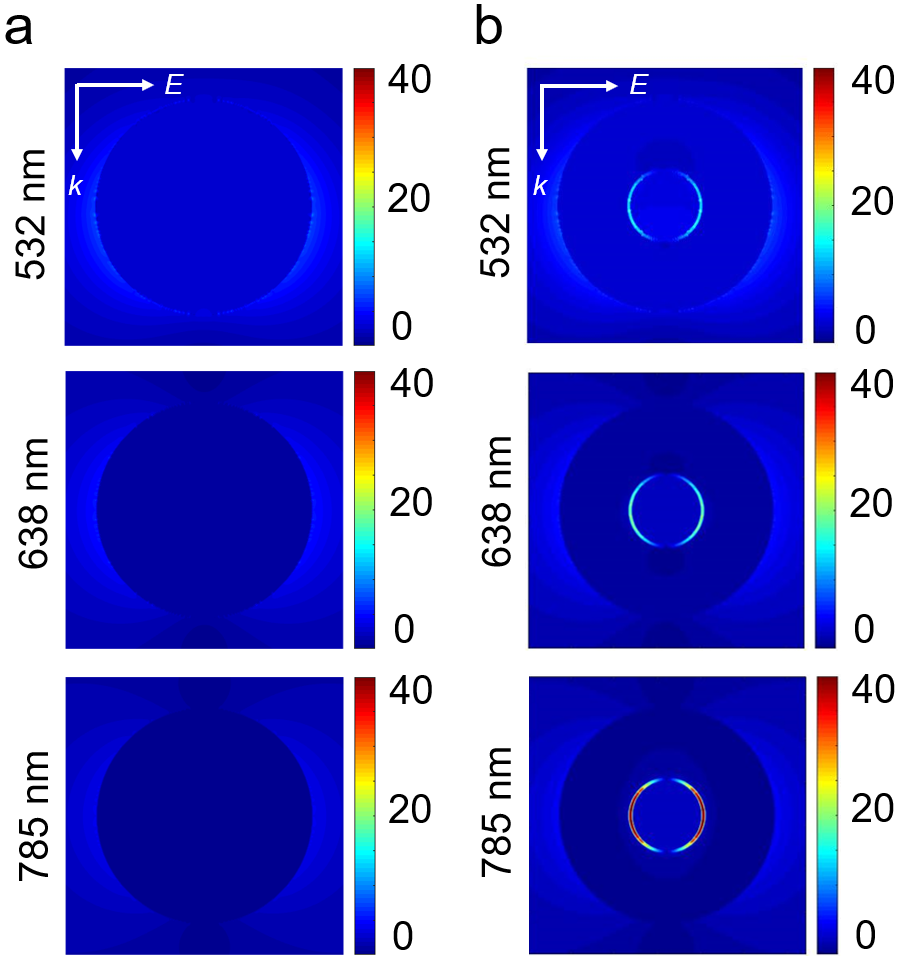


**Figure S11**. FDTD calculated electric field enhancement distribution of (a) a single AuNP (62 nm in diameter) and (b) a single GERT (62 nm in diameter) at excitation wavelengths of 532, 638 and 785 nm. *k* and *E* represent light propagation and polarization direction, respectively.


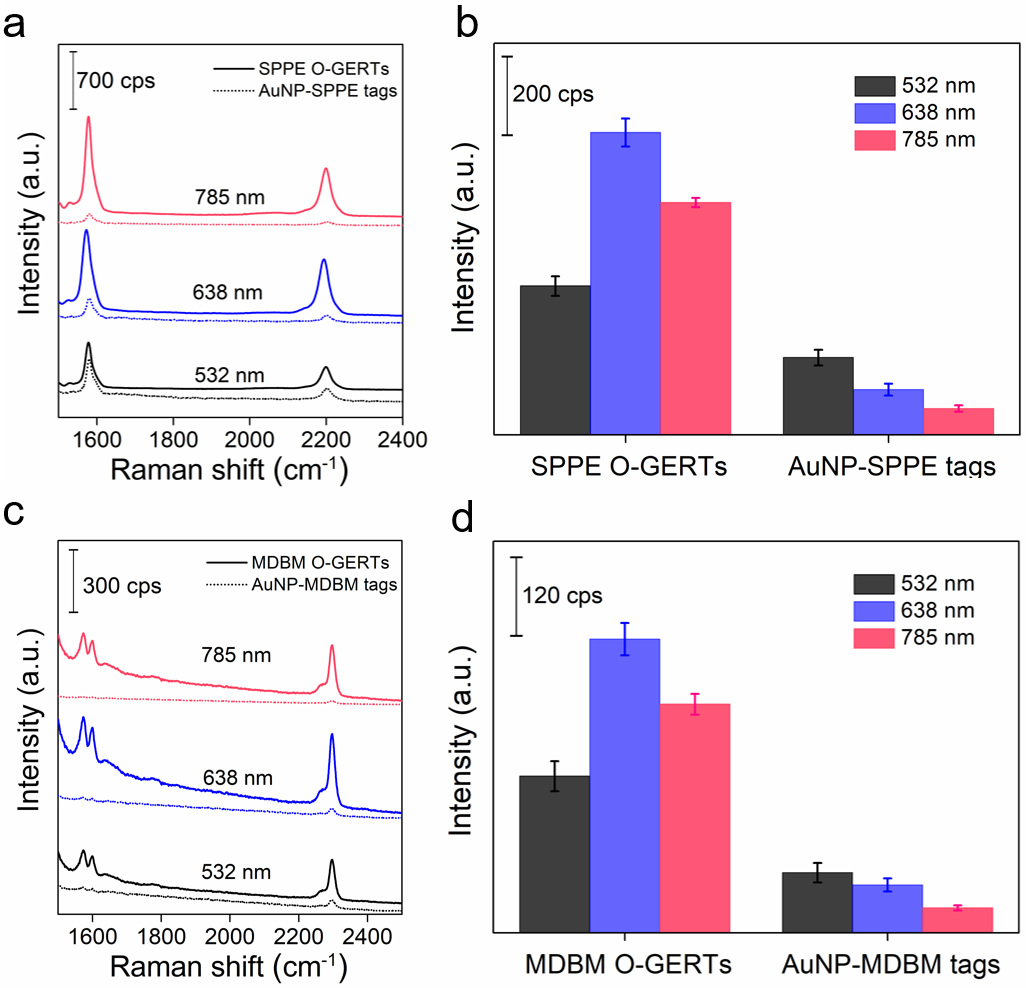


**Figure S12**. Excitation wavelength dependent Raman spectra of O-GERTs and 60 nm sized AuNPs. Raman spectra of (a) SPPE O-GERTs and AuNP-SPPE tags, and (c) MDBM O-GERTs and AuNP-MDBM tags excited by 532, 638 and 785 nm laser. Quantitative comparison of the intensity of Raman band at (b) 2196 cm^-1^ for SPPE O-GERTs and 2201 cm^-1^ for AuNP-SPPE tags, and (d) 2294 cm^-1^ for MDBM O-GERTs and 2297 cm^-1^ for AuNP-MDBM tags in panel a and c. AuNP-SPPE tags and AuNP-MDBM tags represent conventional SERS tags which modified corresponding reporters on the surface of AuNPs.





**Figure S13.** Demonstration of multiplexing capability of four types of O-GERTs with resolvable Raman bands.


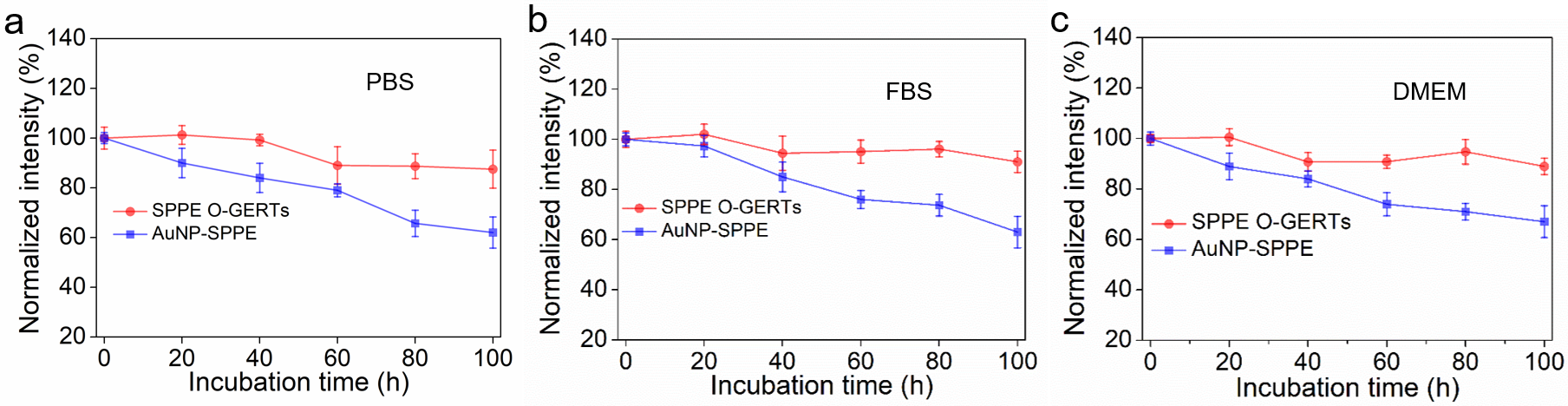


**Figure S14.** Biostability comparison of silent-region signals (2196 cm^-1^) of SPPE O-GERTs and AuNP-SPPE tags in biological fluids of (c) PBS, (d) FBS and (e) DMEM for different times.


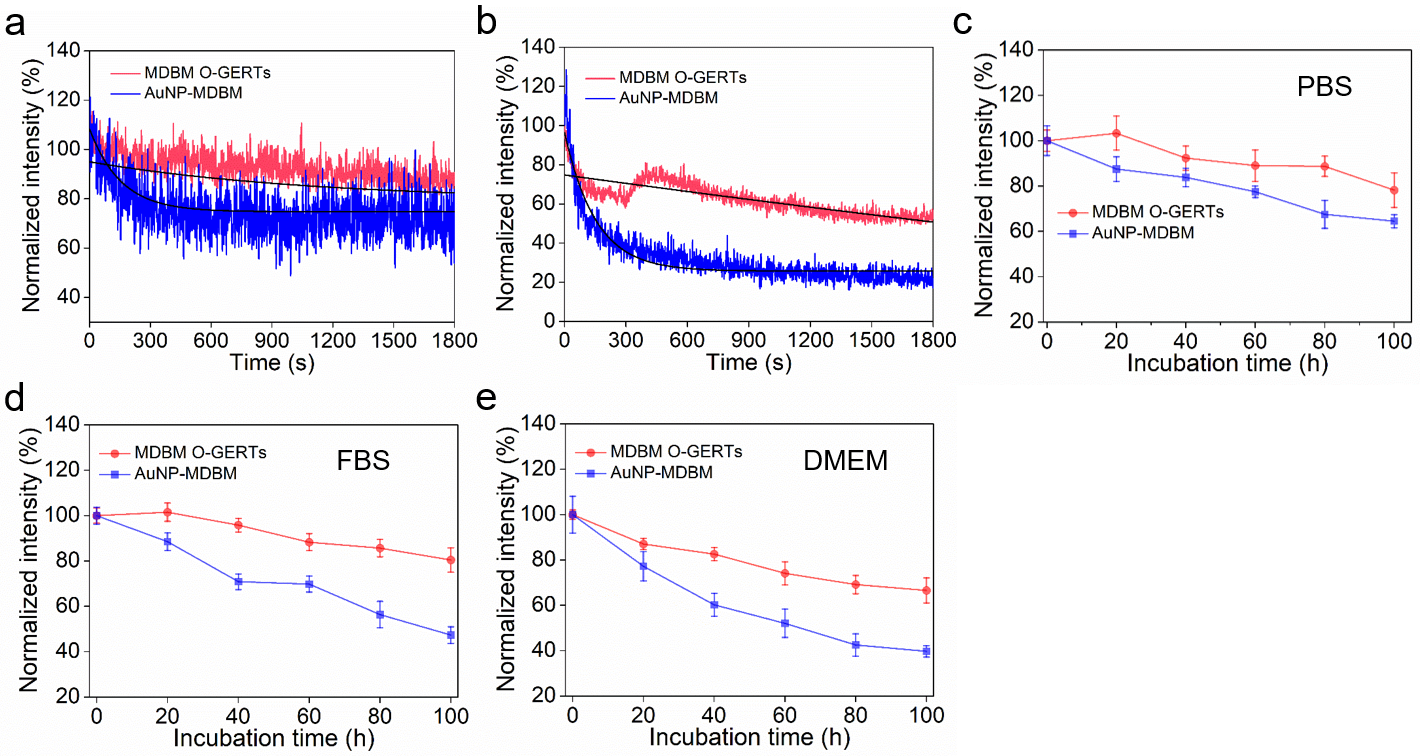


**Figure S15.** Photostability comparison of Raman signals for AuNP-MDBM tags and MDBM O-GERTs under the laser power density of (a) 2.95 × 10^5^ and (b) 1.17 × 10^6^ W/cm^2^. Photostability of both tags were evaluated with integrated peak areas of Raman band at (a) 2294 cm^-1^ for MDBM O-GERTs and (b) 2297 cm^-1^ for AuNP-MDBM tags under continuous laser irradiation with different power densities for 30 min. Through quantitative analysis of photobleaching behaviors of time-resolved SERS trajectories, we found that the photobleaching time constant is 154 s for AuNP-MDBM tags and 1122 s for MDBM O-GERTs under 2.95 × 10^5^ W/cm^2^, while the value is 153 and 11535 s under 1.17 × 10^6^ W/cm^2^, respectively. These results indicate that MDBM O-GERTs show the much higher photostability performance than that of AuNP-MDBM tags. Stability of Raman signals of both tags in biological fluids of (c) PBS, (d) FBS, and (e) DMEM for various durations. The time-dependent signal decrease for AuNP-MDBM tags is likely due to minimized reporter leakage or chemical damage from media [1, 16].


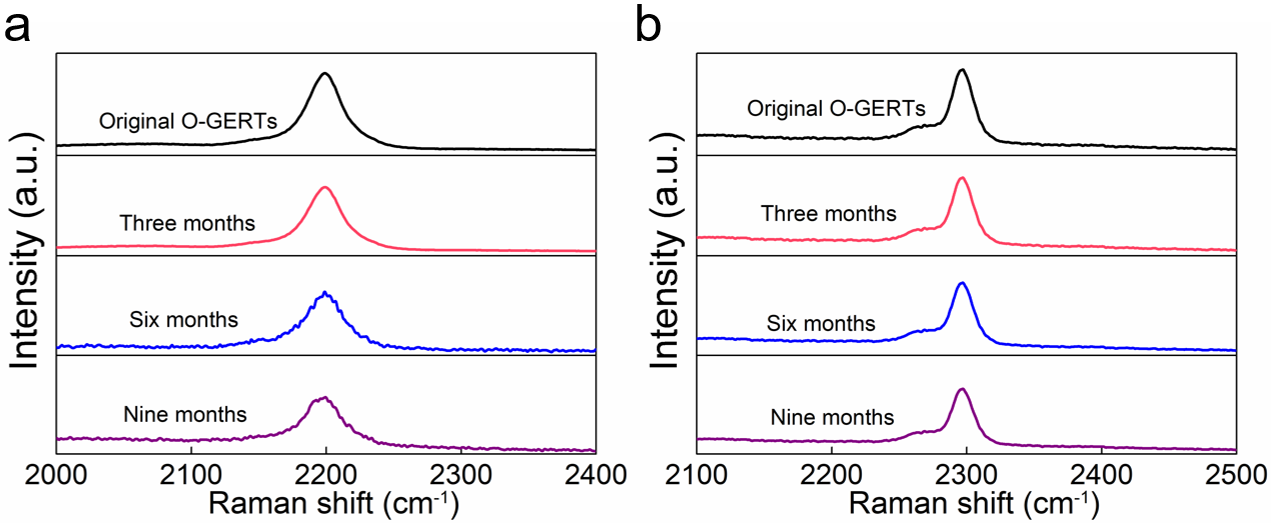


**Figure S16.** SERS spectra of aqueous (a) SPPE O-GERTs and (b) MDBM O-GERTs stored in water for various durations indicating long-term signal stability of both O-GERTs.


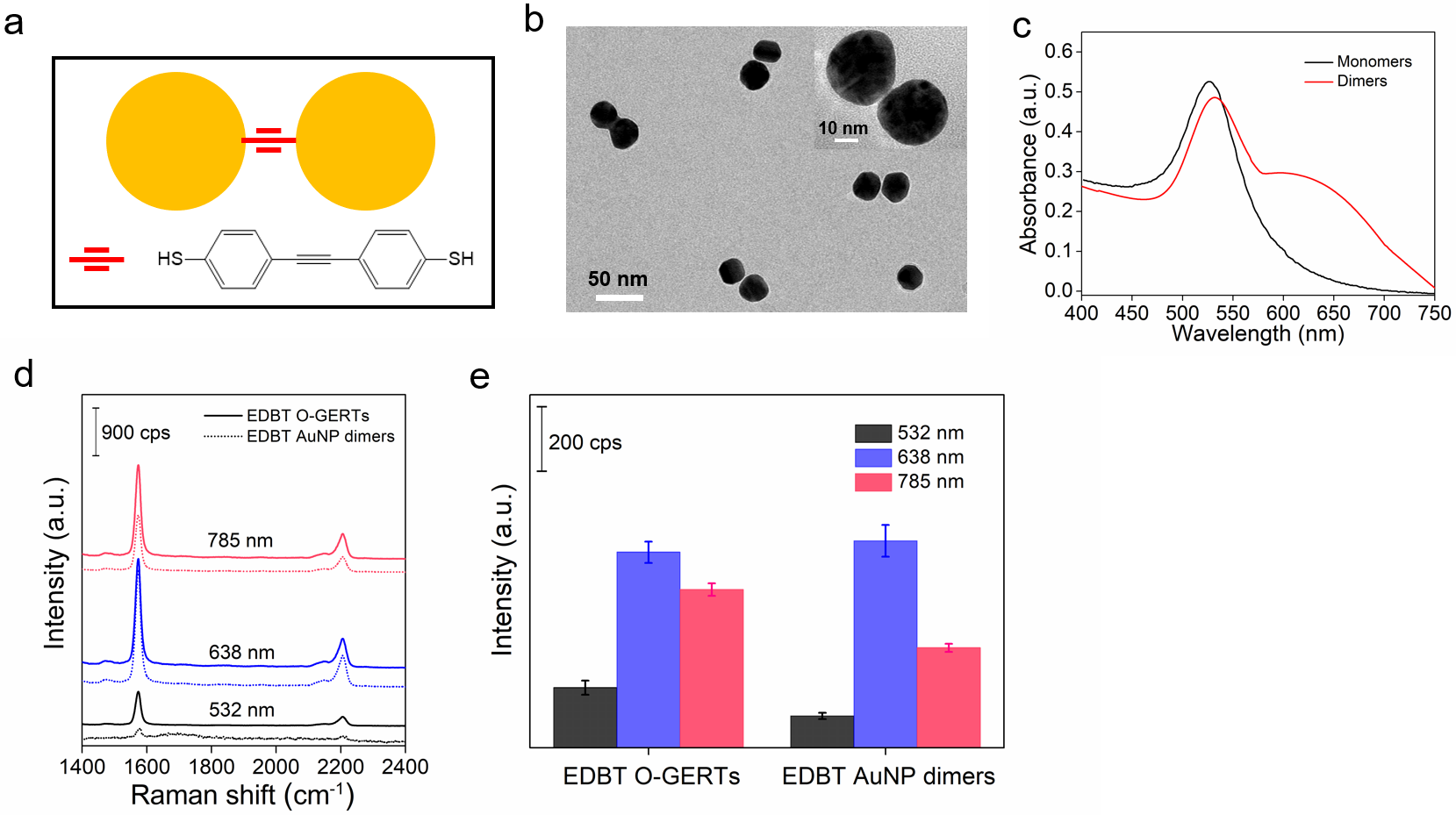


**Figure S17.** (a) Schematic diagram of AuNP dimers linked by EDBT (EDBT AuNP dimers, 60 nm in width). (b) The TEM image of EDBT AuNP dimers. (c) UV-Vis spectra of Au monomers and AuNP dimers. (d) Raman spectra of SPPE O-GERTs and EDBT AuNP dimers excited by 532, 638 and 785 nm laser. (e) Quantitative comparison of the intensity of Raman band at 2205 cm^-1^ for both tags in panel c. EDBT AuNP dimers represent SERS tags with the corresponding Raman reporters decorated in the inter-nanogaps.


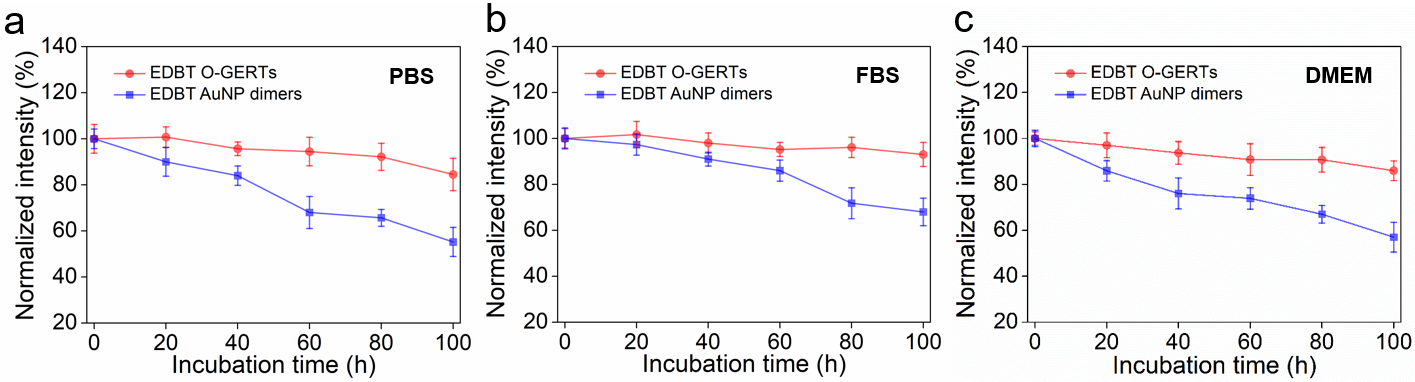


**Figure S18.** Stability of Raman signals (2205 cm^-1^) of EDBT O-GERTs and EDBT AuNP dimers in biological fluids of (c) PBS, (d) FBS, and (e) DMEM for various durations. These results indicate that EDBT O-GERTs show the much higher biological stability performance than that of EDBT AuNP dimers.


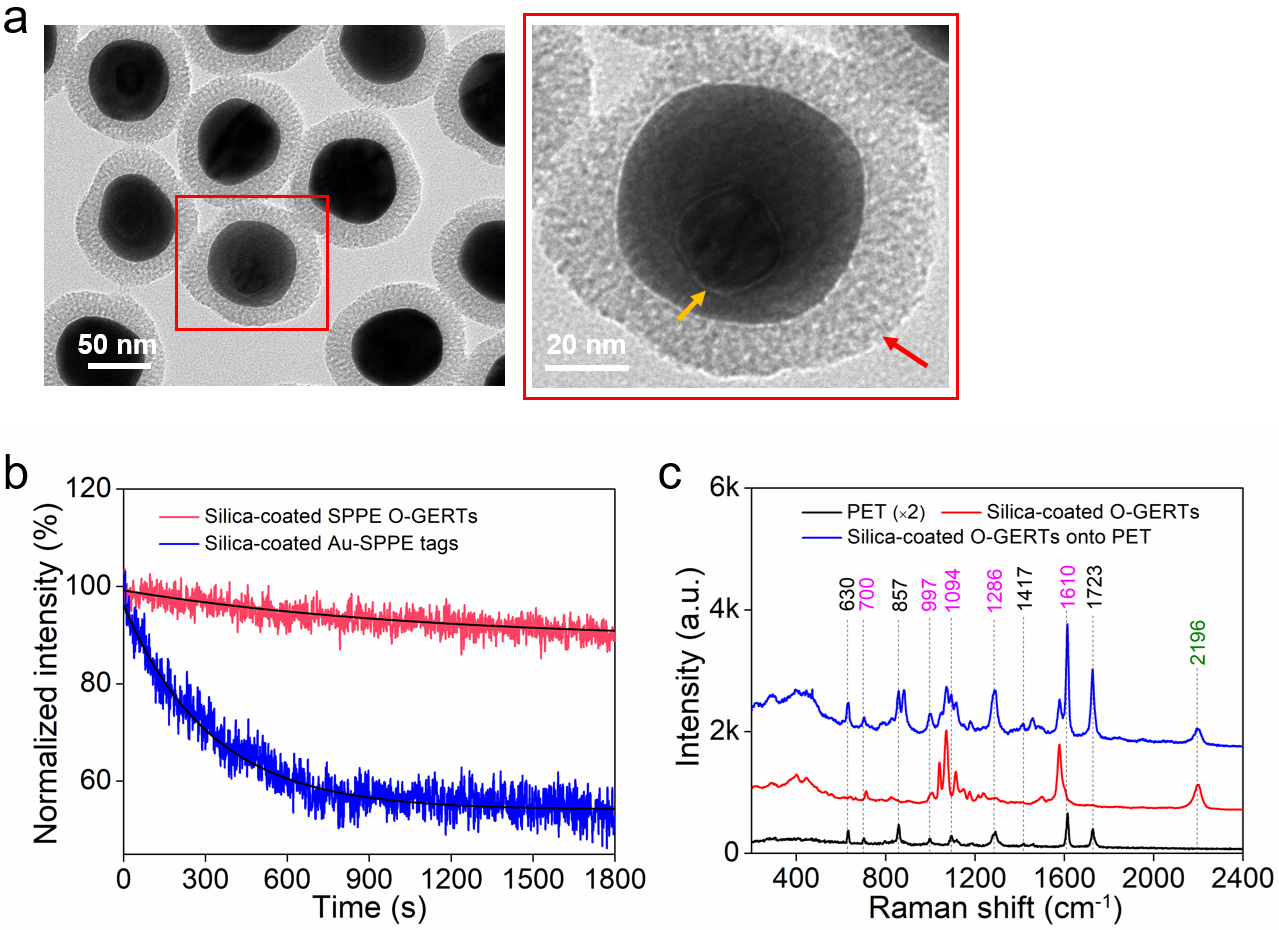


**Figure S19.** (a) TEM image of silica-coated SPPE O-GERTs. The thickness of silica shell is about 15 nm. The yellow and red arrows indicate the internal nanogaps of the O-GERTs and the silica shell, respectively. (b) Photostability of silica-coated SPPE O-GERTs and silica-coated AuNP-SPPE tags under laser power density of 1.17 × 10^6^ W/cm^2^. (c) Raman spectra of silica-coated SPPE O-GERTs onto polyethylene terephthalate (PET). These peaks marked in black, magenta and green are from PET substrates, O-GERTs in the fingerprint region and O-GERTs in the silent region, respectively. It should be note that a thick SiOx layer outside tags may isolate the background from being enhanced by O-GERTs (namely, amplified signal by the plasmonic nanoparticles). However, the Raman signals of the background still exist. To illustrate this, we encapsulated SPPE O-GERTs with a thick silica shell. The thickness of silica shell is ~15 nm and such thickness can sufficiently minimize the background from being enhanced by the near-field effect (typically within a range of 10 nm). Then we measured Raman spectra of silica-coated O-GERTs onto the polyethylene terephthalate (PET) film and the results show that spectral interference still exists in the fingerprint region.


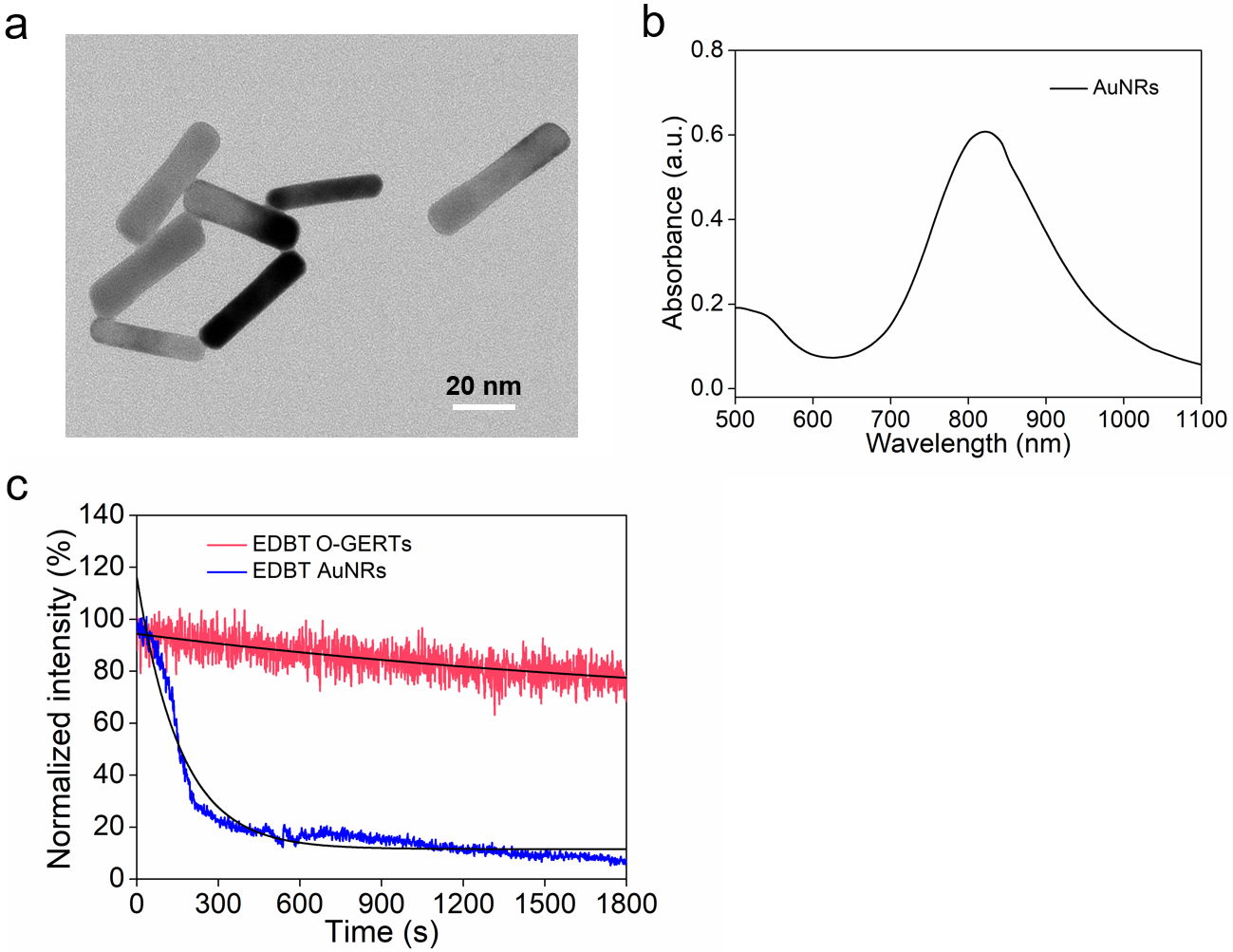


**Figure 20.** (a-b) TEM image and UV-Vis spectra of AuNRs. (c) Photostability of EDBT O-GERTs and EDBT-decorated AuNRs under laser power density of 1.17 × 10^6^ W/cm^2^.

**Table S1.** Summary of photobleaching time constant for seven types of SERS tags.

| **SERS tags** | **Photobleaching time constant (1.17 × 10^6^ W/cm^2^)** |
| --- | --- |
| SPPE O-GERTs | 1063 s |
| silica-coated SPPE O-GERTs | 1099 s |
| AuNP-SPPE tags | 385 s |
| silica-coated Au-SPPE tags | 394 s |
| EDBT AuNP dimers | 473 s |
| EDBT O-GERTs | 2381 s |
| EDBT AuNRs | 160 s |

It should be note that the silica coating can improve the stability of NPs, especially the chemical stability of NPs in different media (*e.g.*, PBS, FBS, DMEM), by isolating the direct contact of Raman reporters with the environment. However, the photostability experiments under same conditions show that the silica shell can slightly improve the photostability of SERS tags (see table above), while the photostability of silica-coated Au-SPPE tags is still much lower than SPPE O-GERTs, highlighting the crucial role of Au shell in O-GERTs to protect the built-in reporters to avoid possible desorption and minimizes photoinduced chemical reactions by isolation from the environment (oxygen, moisture, *etc.*).





**Figure S21**. Raman spectrum of the 96-well plate excited by 785 nm laser.

**Table S2.** Assignment of typical Raman bands from 96-well plates [7, 9, 10].

| Raman bands (cm^-1^) | Assignments |
| --- | --- |
| 392  475 | γ(C-H)  γ(C-C-C) |
| 615 | γ(C-C-C) + ν(C−S) |
| 785 | ν(CH_3_) + γ(CH_2_) |
| 998 | Benzene ring breathing |
| 1028 | Phenyl-ring in-plane deformation |
| 1189 | β(C-H) |
| 1323 | ν(CC) |
| 1442 | δ(CH_2_), CH_2_ scissors bending |
| 1602 | δ(C=C) |





**Figure S22**. Raman spectrum of the PET thin film excited by 785 nm laser.

**Table S3.** Assignment of typical Raman bands from PET [7, 9, 10].

| Raman bands (cm^-1^) | Assignments |
| --- | --- |
| 630  702 | β(C-C-C) + ν(C-S)  γ(CH) + C-C ring bend |
| 858 | ω(C–H) |
| 998 | Phenyl ring breathing |
| 1090 | ν(C-C), skeletal C-C stretches |
| 1117 | ν (C-O) |
| 1188 | δ(C=C) |
| 1287 | δ (COO) |
| 1416 | ν (CO_2_^-^) |
| 1458 | δ(CH_2_) |
| 1612 | δ(C=C) |
| 1727 | δ(C=O) |





**Figure S23.** (a) Raman spectrum of the photo-crosslinked resin excited by 785 nm laser.

**Table S4.** Assignment of typical Raman bands from photo-crosslinked resins [7, 9-11].

| Raman bands (cm^-1^) | Assignments |
| --- | --- |
| 459  568 | γ(C-C-C)  γ(C−C−H) ring bend |
| 630 | γ(C-C-C) |
| 728 | γ(C-H) |
| 842 | -CH_3_ groups (several vibrational modes) |
| 1013 | Benzene ring breathing |
| 1100 | ν(C-C), backbone stretches |
| 1220 | ν(C-O-C) |
| 1297 | ν (C-S) |
| 1442 | δ(CH_2_), CH_2_ scissors bending |
| 1633 | ν(C=C), cis stretch |
| 1723 | δ(C=O), C═O esters |





**Figure S24**. Raman spectrum of three samples of waste water excited by 785 nm laser.


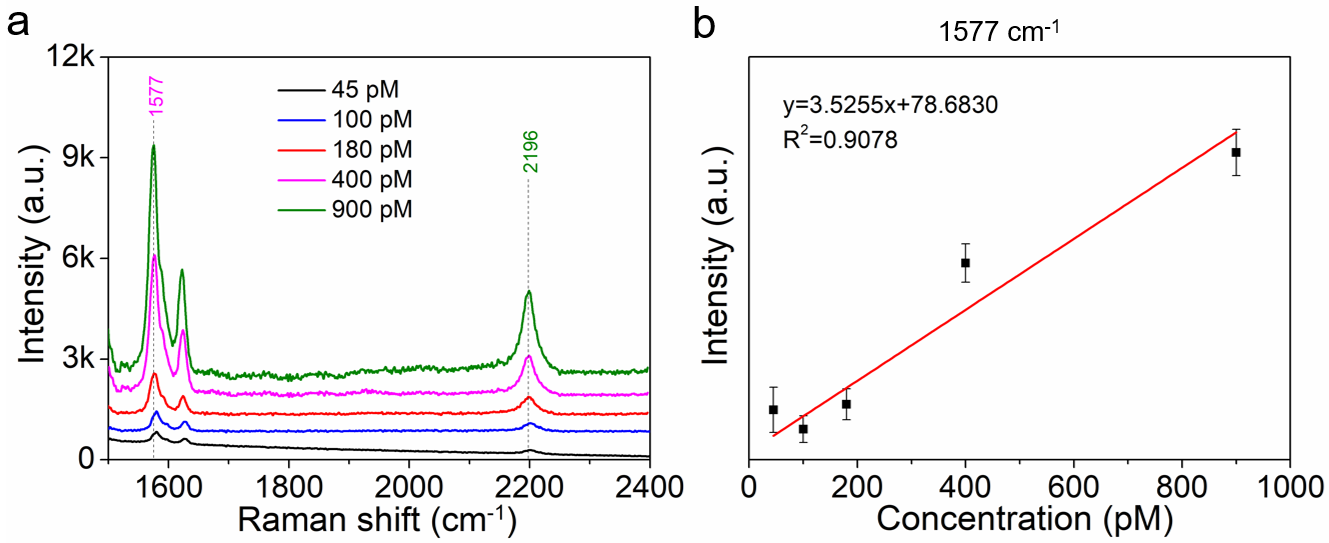


**Figure S25.** (a) Raman spectra of SPPE O-GERTs in the solution of sample 1 from waste water with various concentrations. (b) Calibration curves created by plotting the peak at 1577 cm^-1^ with various concentrations of SPPE O-GERTs in solution of sample 1.





**Figure S26**. Raman spectrum of CB bacteria excited by 785 nm laser.

**Table S5.** Assignment of typical Raman bands from CB bacteria [12, 13].

| Raman bands (cm^-1^) | Assignments |
| --- | --- |
| 416 | Carbohydrates |
| 485 | Carbohydrates |
| 601 | Cholesterol, Ring deformation |
| 681 | δ(COO−), guanine |
| 821 | ν(CN), tyrosin |
| 852 | ω(C–H) |
| 917 | Ring breathing vibration |
| 1054 | Lipid: ν(C-C), phenyl-ring breathing mode (ν_1_) |
| 1266 | Amide III |
| 1463 | δ(CH_2_), saturated lipids |
| 1690 | ν(C=C), protein |





**Figure S27**. Raman spectrum of HeLa cells excited by 785 nm laser.

**Table S6.** Assignment of typical Raman bands from HeLa cells [14, 15].

| Raman bands (cm^-1^) | Assignments |
| --- | --- |
| 437  480 | δ(CSS) and δ(CNC)  Ring twisting |
| 599 | Cytochrome, Ring deformation |
| 800 | RNA, O-P-O |
| 1054 | Lipid: ν(C-C) |
| 1193 | Deoxyribose-phoshpate |
| 1600 | Proteins, ν(C-N), ν(C-C), ρ(CH_3_) |





**Figure S28**. Raman spectrum of the breast cancer tissue excited by 785 nm laser.

**Table S7.** Assignment of typical Raman bands from cancer tissues [14, 15].

| Raman bands (cm^-1^) | Assignments |
| --- | --- |
| 408  599 | Phosphoinositol, CH_2_ bending  Ring deformation |
| 816 | Backbone (RNA) |
| 925 | Peptide: ν(CC) |
| 1001 | Phenyl ring breathing |
| 1065 | C-C chain |
| 1250 | Lipids |
| 1339 | CH_2_, def. |
| 1452 | Deoxyribose, δ(CH_2_) |
| 1670 | Protein: ν(C=C) |


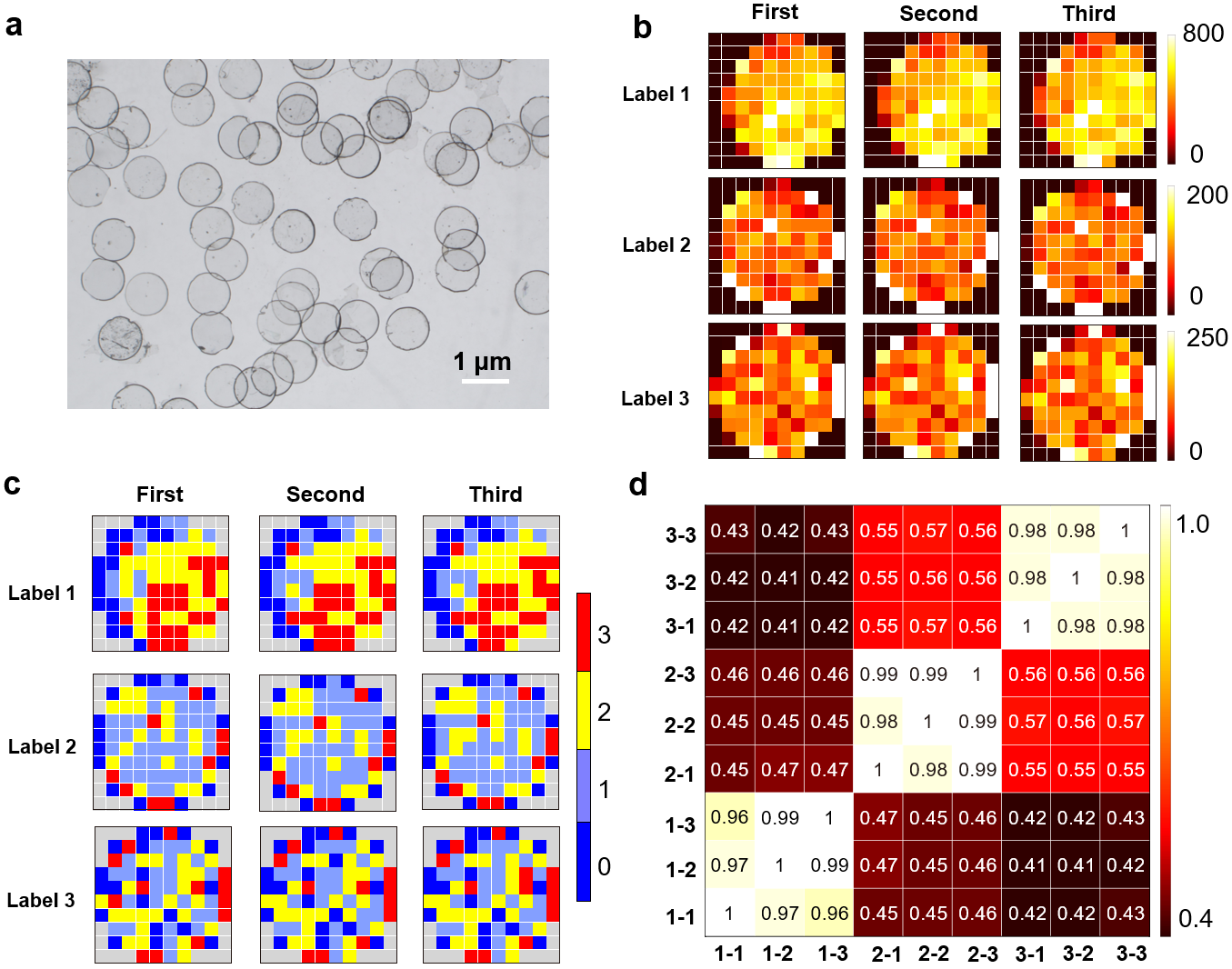


**Figure 29.** Authentication of SPPE O-GERTs based PUF labels. (a) Bright field image of large amounts of disk-shaped labels. (b-c) Raman images with a resolution of 10 × 10 pixels and the corresponding digitized images of three square labels for three repeated measurements. Raman images and digitized codes intensity profiles are derived using the orthogonal band (2196 cm^-1^) of SPPE O-GERTs. (d) Pairwise match of three PUF labels with quaternary encoding of Raman intensity levels at each pixel. 1-1, 1-2 and 1-3 in x-axis represent repeated three measurements from the same label, and 1-1, 2-1 and 3-1 in x-axis represent three different labels measured at the first time, and the color bar shows the similarity index.

# References

[1] J. Li, H. Liu, P. Rong, W. Zhou, X. Gao and D. Liu, "A universal strategy for the one-pot synthesis of sers tags," *Nanoscale*, vol. 10, pp. 8292-8297, 2018.

[2] R. W. Zehner, B. F. Parsons, R. P. Hsung and L. R. Sita, "Tuning the work function of gold with self-assembled monolayers derived from x-[c_6_h_4_-c≡c-]_n_c_6_h_4_-sh (n = 0, 1, 2; x = h, f, ch_3_, cf_3_, and och_3_)," *Langmuir*, vol. 15, pp. 1121-1127, 1999.

[3] Y. Chen, J. Q. Ren, X. G. Zhang, D. Y. Wu, A. G. Shen and J. M. Hu, "Alkyne-modulated surface-enhanced raman scattering-palette for optical interference-free and multiplex cellular imaging," *Anal. Chem.*, vol. 88, pp. 6115-6119, 2016.

[4] H. Di, H. Liu, M. Li, J. Li and D. Liu, "High-precision profiling of sialic acid expression in cancer cells and tissues using background-free surface-enhanced raman scattering tags," *Anal. Chem.*, vol. 89, pp. 5874-5881, 2017.

[5] C. Huang, M. Jevric, A. Borges, et al., "Single-molecule detection of dihydroazulene photo-thermal reaction using break junction technique," *Nat. Commun.*, vol. 8, pp. 15436, 2017.

[6] M. S. Inkpen, Z. F. Liu, H. Li, L. M. Campos, J. B. Neaton and L. Venkataraman, "Non-chemisorbed gold-sulfur binding prevails in self-assembled monolayers," *Nat. Chem.*, vol. 11, pp. 351-358, 2019.

[7] R. A. Alvarez-Puebla, D. S. Dos Santos Junior and R. F. Aroca, "Surface-enhanced raman scattering for ultrasensitive chemical analysis of 1 and 2-naphthalenethiols," *Analyst*, vol. 129, pp. 1251-1256, 2004.

[8] T. Fernandes, S. Fateixa, H. I. S. Nogueira, A. L. Daniel-da-Silva and T. Trindade, "Dendrimer-based gold nanostructures for sers detection of pesticides in water," *Eur. J. Inorg. Chem.*, vol. 2020, pp. 1153-1162, 2020.

[9] F. Madzharova, Z. Heiner and J. Kneipp, "Surface-enhanced hyper raman spectra of aromatic thiols on gold and silver nanoparticles," *J. Phys. Chem. C*, vol. 124, pp. 6233-6241, 2020.

[10] S. R. Smith and J. Lipkowski, "Guided assembly of two-dimensional arrays of gold nanoparticles on a polycrystalline gold electrode for electrochemical surface-enhanced raman spectroscopy," *J. Phys. Chem. C*, vol. 122, pp. 7303-7311, 2018.

[11] H. Qu, M. Yu, W. Du, L. Xu, W. Lyu and F. Shen, "Slip molding for precision fabrication of microparts," *Langmuir*, vol. 36, pp. 585-590, 2020.

[12] N. E. Mircescu, H. Zhou, N. Leopold, et al., "Towards a receptor-free immobilization and sers detection of urinary tract infections causative pathogens," *Anal. Bioanal. Chem.*, vol. 406, pp. 3051-3058, 2014.

[13] S. A. Strola, J. C. Baritaux, E. Schultz, et al., "Single bacteria identification by raman spectroscopy," *J. Biomed. Opt.*, vol. 19, pp. 111610, 2014.

[14] S. Hong, L. Lin, M. Xiao and X. Chen, "Live-cell bioorthogonal raman imaging," *Curr. Opin. Chem. Biol.*, vol. 24, pp. 91-96, 2015.

[15] C. Krafft, I. W. Schie, T. Meyer, M. Schmitt and J. Popp, "Developments in spontaneous and coherent raman scattering microscopic imaging for biomedical applications," *Chem. Soc. Rev.*, vol. 45, pp. 1819-1849, 2016.

[16] D. K. Lim, K. S. Jeon, J. H. Hwang, et al., "Highly uniform and reproducible surface-enhanced raman scattering from DNA-tailorable nanoparticles with 1-nm interior gap," *Nat. Nanotechnol.*, vol. 6, pp. 452-460, 2011.
